# Capsaicin: A Two-Decade Systematic Review of Global Research Output and Recent Advances Against Human Cancer

**DOI:** 10.3389/fonc.2022.908487

**Published:** 2022-07-13

**Authors:** Tomi Lois Adetunji, Femi Olawale, Chijioke Olisah, Ademola Emmanuel Adetunji, Adeyemi Oladapo Aremu

**Affiliations:** ^1^ Unit for Environmental Sciences and Management, North-West University, Potchefstroom, South Africa; ^2^ Nano-Gene and Drug Delivery Group, Discipline of Biochemistry, School of Life Sciences, College of Agriculture, Engineering and Science, University of KwaZulu-Natal, Durban, South Africa; ^3^ Department of Botany and Institute for Coastal and Marine Research, Nelson Mandela University, Port Elizabeth, South Africa; ^4^ Department of Molecular and Cell Biology, University of Cape Town, Cape Town, South Africa; ^5^ Indigenous Knowledge Systems Centre, Faculty of Natural and Agricultural Sciences, North-West University, Mmabatho, South Africa; ^6^ School of Life Sciences, College of Agriculture, Engineering and Science, University of KwaZulu-Natal, Durban, South Africa

**Keywords:** anticancer, bibliometrics, cytotoxicity, TRPV 1, vanilloid

## Abstract

Capsaicin (8-methyl-N-vanillyl-6-nonenamide) is one of the most important natural products in the genus *Capsicum.* Due to its numerous biological effects, there has been extensive and increasing research interest in capsaicin, resulting in increased scientific publications in recent years. Therefore, an in-depth bibliometric analysis of published literature on capsaicin from 2001 to 2021 was performed to assess the global research status, thematic and emerging areas, and potential insights into future research. Furthermore, recent research advances of capsaicin and its combination therapy on human cancer as well as their potential mechanisms of action were described. In the last two decades, research outputs on capsaicin have increased by an estimated 18% per year and were dominated by research articles at 93% of the 3753 assessed literature. In addition, anti-cancer/pharmacokinetics, cytotoxicity, *in vivo* neurological and pain research studies were the keyword clusters generated and designated as thematic domains for capsaicin research. It was evident that the United States, China, and Japan accounted for about 42% of 3753 publications that met the inclusion criteria. Also, visibly dominant collaboration nodes and networks with most of the other identified countries were established. Assessment of the eligible literature revealed that the potential of capsaicin for mitigating cancer mainly entailed its chemo-preventive effects, which were often linked to its ability to exert multi-biological effects such as anti-mutagenic, antioxidant and anti-inflammatory activities. However, clinical studies were limited, which may be related to some of the inherent challenges associated with capsaicin in the limited clinical trials. This review presents a novel approach to visualizing information about capsaicin research and a comprehensive perspective on the therapeutic significance and applications of capsaicin in the treatment of human cancer.

## 1 Introduction

Capsaicin (8-Methyl-N-vanillyl-trans-6-nonenamide, C_18_H_27_NO_3_) is a homovanillic acid abundant in *Capsicum* species (pepper) fruits (1, 2). Its structure (**Supplementary Figure 1**) was ascertained in 1919, following its isolation in 1846 (3, 4) and chemical synthesis in 1930 (5). The compound has two geometric (cis-trans) isomers but naturally occurs as a trans-isomer (6). This lipophilic compound is the main bioactive constituent of peppers and is responsible for the tissue irritation and characteristic burning consequences (pungency) of peppers (1, 2). It accounts for about 70% of the alkaloid group called capsaicinoids. Other analogs are dihydrocapsaicin (second most abundant, representing ca. 22%), nordihydrocapsaicin (ca. 7%), and compounds such as homocapsaicin, homodihydrocapsaicin and norcapsaicin produced in lower quantities (2). Capsaicin constitutes a key ingredient of self-protection products (e.g., oleoresin capsicum spray), spicy foods in various cultures around the world, and its concentration may be more than 65% in cosmetic, herbal supplements, and other health care products (7).

Capsaicin content is high in pepper fruit placenta, which holds the seeds (3, 8). The ovary and fruit–tip contain the highest capsaicin content, while the seeds have the lowest concentration (9). Capsaicin levels may increase in pepper when subjected to controlled-stress conditions (10, 11). Due to its broad applicability, there have been extensive studies aimed at enhancing capsaicin production. For instance, capsaicin production has been improved by enzyme-catalyzed (12), chemical (13), and *in vitro* syntheses (14, 15) as well as improving pepper cultivation (6, 16). Its biosynthesis by fatty acid metabolism and phenylpropanoid pathways (**Supplementary Figure 2**) has been described by several authors (10, 11, 17).

There is increasing interest in using capsaicin as a therapeutic alternative for different diseases (18, 19) due to its pleiotropic pharmacological effects on various physiological systems, with an emphasis on pain as well as neuroscience, cardiovascular, respiratory, cancer, and urinary systems studies (20). In terms of the pharmacokinetics, capsaicin has high oral bioavailability and skin absorption (21), making its topical application effective in various musculoskeletal or neuropathic pain conditions such as arthritis (22), shingles (23), vasomotor rhinitis (24), vasogenic facial pain (25). It is also used for treating urinary incontinence, chronic kidney disease-associated pruritus, and postoperative nausea and vomiting in acupoint therapy (3). Other beneficial bioactivities of capsaicin, including analgesic, anesthetic, anti-apoptotic, anti-inflammatory, anti-obesity, antioxidant, neuroprotective effects (1, 26, 27), enhanced energy metabolism (28), gastroprotective (29), and anticarcinogenic properties (7, 30) have also been reported. However, capsaicin may also function as a carcinogen or co-carcinogen (7, 31, 32).

Understanding capsaicin research from global perspectives over an extended time is crucial. Although several studies have been published on capsaicin applications, bioactivities, and many other capsaicin-related topics (33–35), none of these studies explored the scientometric approach to critically assess its progress and current direction in scientific research. Bibliometrics is a valuable tool for evaluating research trends within a subject area, thus providing insight into extensively researched themes and identifying research needs to inform action (36).

Web of Science Core Collection indexing coverage search gave more than 19,000 publications about capsaicin from 1991 to 2021. A recent review also showed that out of over 10,000 capsaicin-related publications from 2010 to 2020, the anticancer effect was the most investigated, accounting for ca. 26% (2). Hence, this review aimed to provide a systematic review of global research output and recent advances in capsaicin application against human cancer in the last two decades. The in-depth analysis of retrieved publications provided an overview of explored themes, research progression, as well as insights and future perspectives needed to enrich the knowledge domain on the compound.

## 2 Methodology

### 2.1 Data Gathering and Selection Criteria

Data used for the bibliometric survey was retrieved according to the procedure described in our previous articles (36, 37)(**Supplementary Figure 3**). Concisely, published articles on “Capsaicin” were retrieved from the Web of Science (WoS) Core Collection and Scopus databases. The former database was chosen because it contains a high volume of biological and physical sciences literature (38, 39), while Scopus is considered the largest citation and abstract source of global research outputs (40). In the WoS database, the search term “Capsaicin” was used to retrieve records in the “Title” module from January 1, 2001 to December 31, 2021. Only document types such as “Article”, “Review”, “Book Chapter”, and “Editorial” were searched. The search yielded 2914 records. Other document types such as “Proceeding Paper,” “Letters,” “News Items,” “Corrections,” “Early Access,” “Retracted Publications,” and “Publication with Expression of Concern,” were excluded from the search because these are often pre- or post-publication data. For Scopus, a total of 3261 records were identified using the search term “Capsaicin” on the “Article Title” search field. Only records such as “Article”, “Review”, “Editorial”, and “Book Chapter” that satisfied the selection criteria were included. Other records such as “Conference Paper,” “Note,” “Letter,” “Erratum,” “Short Survey,” and “Retracted” were excluded. Records from both databases were downloaded in Bibtex file format and uploaded in RStudio (Version 1.1.463, 2009–2018) for statistical processing. The search and data retrieval were conducted on January 31, 2022. Bibliometric library and packages were installed on the RStudio and used to analyse all bibliometric indicators (articles produced per year, most used keywords, most productive authors, and countries based on number of publications and citations). Duplicate records from both databases were merged as one using R commands. Codes for all bibliometric indicators were obtained from https://www.bibliometrix.org/vignettes/Introductiontobibliometrix.html. Keyword visualization was done on VOSviewer (version 1.6.15, 2009 –2020). Information on recent research advances of capsaicin against human cancer was obtained from relevant articles published in the last two decades in different databases, including Google Scholar, WoS, and Scopus. Chemical structures were drawn with ChemDraw.

## 3 Results and Discussion

### 3.1 Descriptive Analysis of Scientific Production and Annual Publication Trends

A total of 3753 scientific documents met the inclusion criteria in the merged databases (WoS and Scopus) across the 20 years from 2001 to 2021. The entire capsaicin-related research was divided into five groups according to the type of document: articles (3460) representing 93% of the total, followed by reviews (195, 5%), editorial materials (43, 1.2%), book chapters (54, 1.4%), and books (1). Documents were retrieved from 1385 journals and included 6920 author keywords (DE) and 12586 keywords plus (ID). Except for 115 single-authored publications, all authors (10113) published multiple-author articles with an average author-per-document and document-per-author of 2.69 and 0.371, respectively (**Supplementary Table 1**).

**Figure 1:**
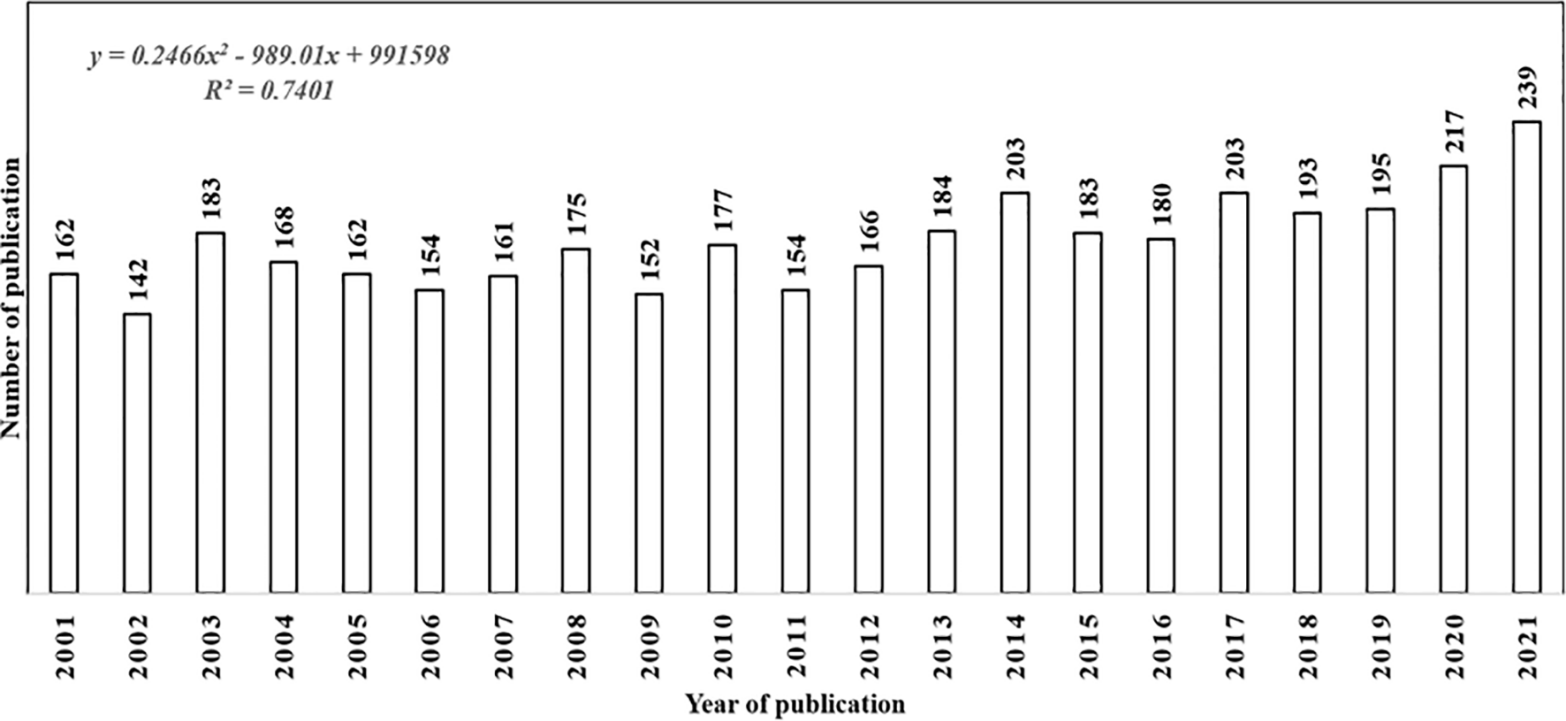
Annual number of publications relating to capsaicin research in the period 2001–2021.

**Figure 1** presents the annual scientific production and trends in capsaicin research from 2001 to 2021. Given its wide display of biological effects and therapeutic significance since its identification, capsaicin has been the target of extensive research (41). The number of articles related to capsaicin research increased from 162 in 2001 to 239 in 2021, with an annual growth rate of 18.88%. From 2001 to 2017, it was observed that there were fluctuations in publication productivity. However, in 2018, the publication productivity of capsaicin research increased steadily up until 2021. The relationship between the publication year and the number of publications fitted into the polynomial model showed a strong positive correlation r^2^ value of 0.956. This result, together with other statistical measures, such as Kolmogorov-Smirnoff goodness-of-fit (0.719) and β-coefficient (2. 446), suggests that there could be an increasing trend in publication productivity with persistent investigations.

Regarding the publication language, the majority of the articles were published in English (98%), however, some articles were also published in German (0.5%), French (0.3%), and Spanish (0.2%). Other languages, such as Chinese, Portuguese, Turkish and Polish, occurred in lower frequencies. Evidently, capsaicin research topics are rising trends in scientific research, and several researchers across the globe are actively contributing to the field. The diversity of research topics on capsaicin is evident in the distribution of different publications in science-based subject areas, including neurosciences, pharmacology, biochemistry and/or molecular biology, physiology, chemistry, anaesthesiology, cell biology, food science and technology, science technology and gastroenterology (hepatology). This underscores the recognition of capsaicin as a promising drug candidate to be developed as a primary treatment therapy for several ailments (41).

### 3.2 Keyword Analysis and Thematic Areas

**Figure 2:**
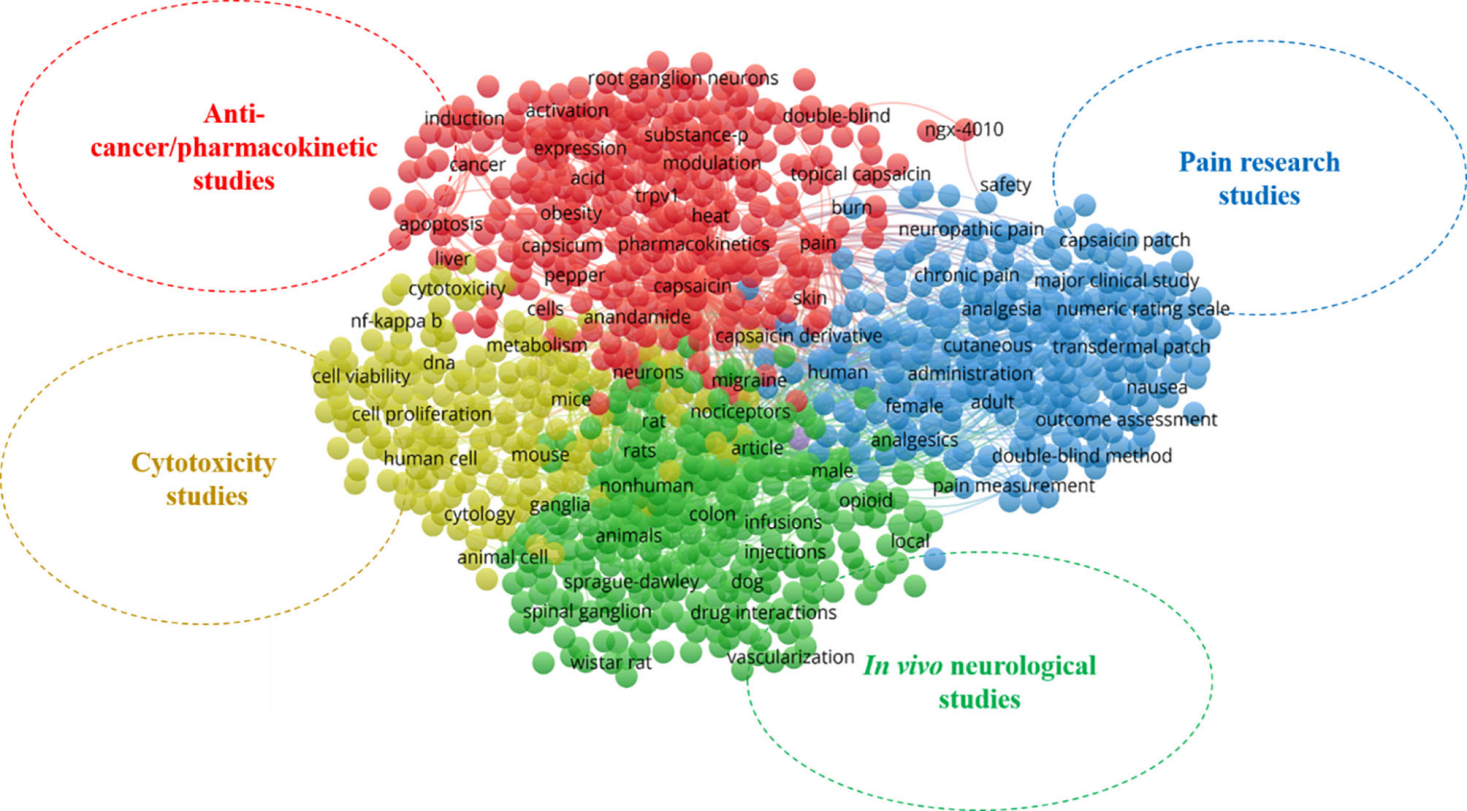
Thematic areas and network visualization of keyword co-occurrence map on capsaicin publications.

In scientometric analysis, keywords in publications are generally accepted as representations for obtaining insights into the thematic area of the research (42). Here, the top 20 most relevant keywords [author’s keywords (DE) and keyword-plus (ID)] in capsaicin research were recorded (**Supplementary Table 2**). To evaluate the thematic areas of capsaicin-related publications, an analysis of the co-occurrence network of keywords associated with capsaicin research was done for the period under study. Four keyword clusters can be interpreted as the thematic areas in the study domain, where each cluster represents a thematic domain (**Figure 2**). The terms enclosed in different coloured circles in a cluster represent the most frequently used keywords. Lines between terms show the frequency of occurrence in literature.

In **Figure 2**, the blue cluster (cluster 1) represents the first thematic area. It focuses on pain research studies on capsaicin with different keywords such as pain measurement, analgesics, neuropathic pain, and chronic pain. Indeed, capsaicin is an incontestably thrilling molecule and remains a valued drug for easing pain (41). Currently, capsaicin is in the third phase (phase III) of clinical trials as an analgesic agent for musculoskeletal, chronic/acute, arthritis, neuropathic, and postoperative pains (41, 43).

The green cluster (cluster 2), with keywords such as animals, rats, dogs, Wistar rat, Sprague- Dawley, neurons, nociceptors, spinal ganglions, and ganglia represents *in vivo* neurological thematic area. This cluster covers publications that document the application of *in vivo* models to study the mechanism by which capsaicin exerts its many therapeutic effects on the human nervous system. The utilization of capsaicin as a therapeutic agent stems from its relatively selective capacity to excite and or cause neuroinhibitory action (capsaicin desensitization) of a subpopulation of afferent neurons [transient receptor potential channel vanilloid (TRPV1)] receptors, which reduces the number of nerve fibers that respond to painful stimuli (44, 45). Hence, extensive studies have been done in this thematic area over the last two decades.

In the yellow cluster (cluster 3), keywords such as cytotoxicity, human cells, cell viability, cytology, and animal cell focusing on cytotoxicity study thematic area are grouped. Although, with proper dosage, capsaicin has demonstrated several health-promoting effects, high doses of capsaicin can cause various acute physiological responses (e.g., burning sensation), activate inflammation, and induce cytotoxicity in various cells (46). Thus, there has been significant research on the cell toxicity of capsaicin to ensure the most effective dosage in treating different ailments. Apart from the side effects of capsaicin prompting cytotoxicity studies, capsaicin has shown wide applicability against several types of cancer (30) through the induction of apoptosis and arrest of cell cycle progression (47). Hence, studies to explore the effects of capsaicin on cancer cell lines have persisted.

The red cluster (cluster 4) depicts the pharmacokinetics and anticancer thematic area of capsaicin research and includes different keywords such as modulation, expression, induction, activation, TRPV1, cancer and apoptosis. Over the last two decades, there have been several publications investigating the anticancer, mechanisms of action, pharmacokinetics, and pharmacodynamics of capsaicin since this compound exerts many pathways in its mode of action against different ailments.

### 3.3 Publication Sources and Topmost Journals

Research outputs on capsaicin were published in 1385 primary reference works such as conference proceedings, journals, books, and letters. The top 20 most productive journals in capsaicin-related research are recorded in **Supplementary Table 3**. Based on the compiled data, three publishers − Elsevier, Wiley Blackwell, and Lippincott Williams & Wilkins were identified as the top publishers, with seven Elsevier journals, two Wiley Blackwell journals, and two Lippincott Williams & Wilkins journals. Different publishers publish the remaining journals across the globe. The most productive journal in terms of the number of articles was “*Pain*” with 363 articles, followed by “*European Journal of Pharmacology*” (78 articles), “*Neuroscience Letters*” (63 articles), “*Neuroscience*” (56 articles), and “*Brain Research*” (55 articles). *Pain*, which had the highest number of articles on capsaicin research, is one of the top journals devoted to publishing original research articles that deal with the nature, mechanisms, and treatment of pain. One of the major therapeutic applications of capsaicin is its topical use in treating pain. Capsaicin studied in several *in vitro* and *in vivo* models as well as clinical trials has shown therapeutic effectiveness against acute and chronic pains and has been approved as a topical treatment of neuropathic pain (48). The mechanism of action of its pain-relieving effect has been attributed to the ability of capsaicin to cause reversible defunctionalization or desensitization of sensory nerve endings of substance P and by reducing the density of epidermal nerve fibers (49). The increasing interest of researchers in understanding capsaicin’s effects and the mechanisms of action on chronic pain may account for the high number of articles on capsaicin published in *Pain* journal. In terms of impact factor, the *British Journal of Pharmacology* (IF 8.739) was the most influential, followed by *Pain* (IF 6.96), *Journal of Neuroscience* (IF 6.167), *Journal of Pain* (IF 5.820), and *Journal of Agricultural and Food Chemistry* (IF 5.279).

### 3.4 Leading Authors and Citation Analysis

An important aspect of bibliometrics is the contribution of authors toward a research topic. Citation indicators or metrics, especially the H-index (an author-level metric that measures both the productivity and citation impact of the publications), are generally being used in the context of research evaluation (50). Several studies have shown that the H-index is correlated with the total number of citations and publications (51, 52). The present study used different citation metrics such as the number of articles, H-index, g-index, and total citations to identify the top 20 leading authors (authors who have contributed more than 20 publications) in capsaicin research over the last two decades (**Supplementary Table 4**). The top leading author’s productivity is shown in **Supplementary Figure 4**, where embedded circles represent the total number of articles and total citations for articles published in a particular year.

The results of the analysis show that the top five most productive authors are Lee, J. (46 articles), Lee, S. (45 articles), Wang, X (43 articles), Wang, Y. (43 articles) and Zhang, Y. (42 articles). As for the relative impact of the publication in terms of citations, Anand, P. (3166 citations), Lee, S. (1763 citations), Wang, X. (1282 citations), Wang, J. (1124 citations) and Wang, H. (1075 citations) were the most influential in the period considered.

### 3.5 Most-Cited Publications

The number of citations an article receives has been employed as a marker of its influence on the research community in that subject area (53). We identified the 20 most cited articles in the field of capsaicin research during the 20 years study period (**Supplementary Table 5**) and their association with the clusters identified in **Figure 2** (the thematic areas of capsaicin research). The listed 20 most-cited articles gave insight into the important articles and thematic areas that had impacted capsaicin research within and beyond the subject area. These articles were all published by authors from developed countries and were co-authored collaborations except Amadesi (54) and Ghilardi (55). These top-cited articles received between 240 and 1048 citations, and only four articles were cited more than 400 times. The most cited article was “Bradykinin and nerve growth factor release the capsaicin receptor from PtdIns(4,5)P2-mediated inhibition” by Chuang et al. (56), published in *Nature* with 1048 citations. By evaluating the listed articles (**Supplementary Table 5**) in relation to the thematic clusters in **Figure 2**, it appears that research focusing on the pharmacokinetics/pharmacodynamics of capsaicin (cluster 4) has made the greatest contributions, as six of the 20 most cited publications are associated with this cluster.

### 3.6 Leading Countries and Collaboration Networks Between Countries

**Figure 3:**
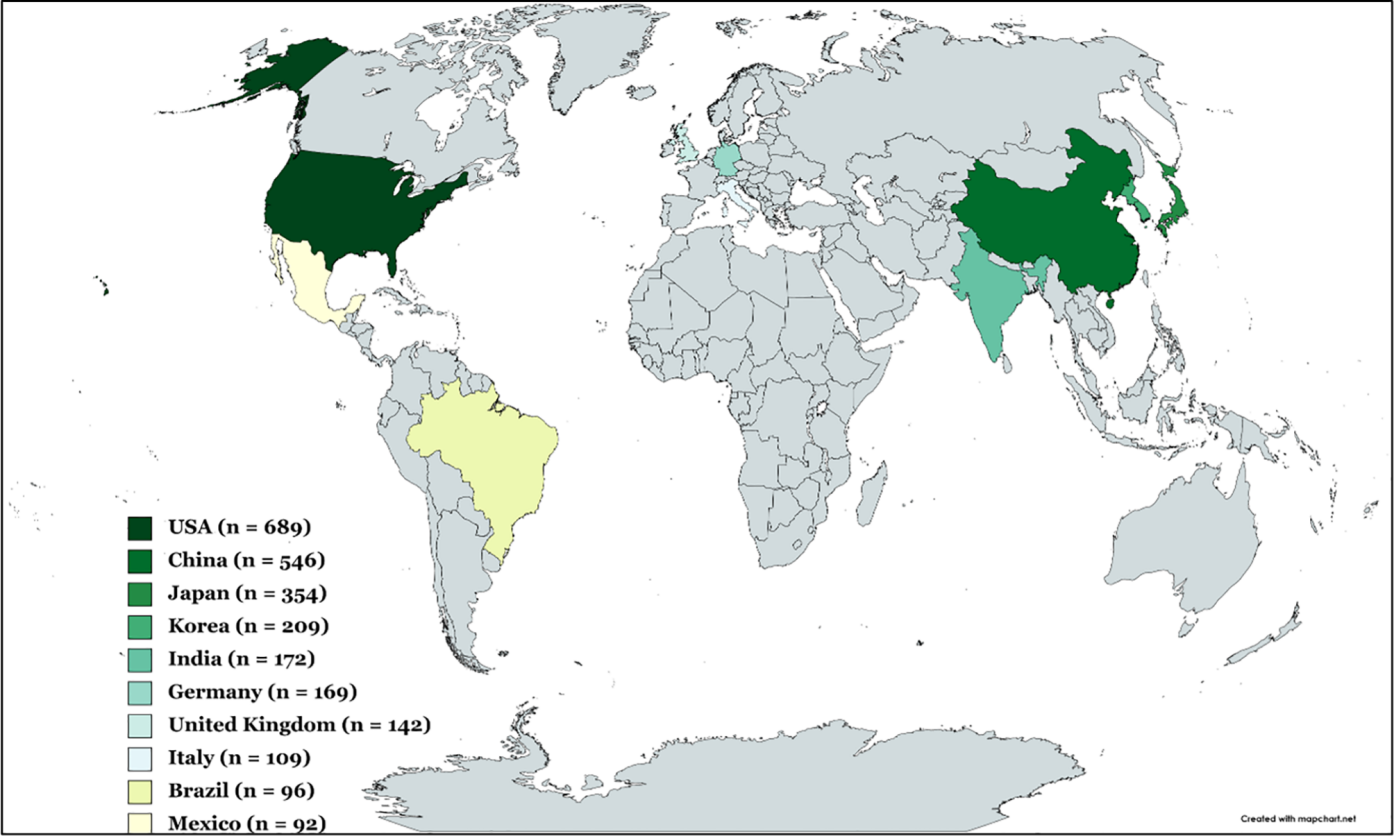
Heat map of the top 10 leading countries based on capsaicin publications from 2001 to 2021. Grey colour shadings signify countries outside the top 10.

The heat map of the 10 top leading countries that have contributed more than 30 publications over the 20 years study period was shown in **Figure 3**. Additional information given in **Supplementary Table 6**, showed the number of articles, citations, average article citations, single country publications, and multiple country publications. The highest publication metrics were from the United States (689 publications), China (546 publications), Japan (354 publications), North Korea and South Korea (209 publications), and India (172 publications), making them the top five leading countries in terms of the number of publications. Of the top leading 20 countries, the dominance of European countries and, to a lesser degree, Asian countries was striking, while publications originating from North America, South America, and Oceania were less prevalent. Within Asia, the majority of the publications originated from the far East, and China, Japan, North and South Korea, and India were recognized as significant contributors to capsaicin research. In Europe, Germany, United Kingdom, and Italy were significant contributors to capsaicin publications. Europe has been recognized as the centre of global science and research since the beginning of the 20^th^ century as scientists from relatively rich European countries are heavily funded (57). This could account for the relatively high contributions of capsaicin publications from Europe. In terms of citations, the top three countries — the US (28869 total citations), China (8621 total citations), and Japan (8578 total citations) corresponded with the top three productive countries. Other countries with relatively high citations were the United Kingdom (7857 total citations) and North and South Korea (5813 total citations).

To assess the international collaboration network on capsaicin research among the 20 leading countries, a network visualization map was constructed (**Supplementary Figure 5**). Generally, more productive countries (in terms of publications and citations) also have more collaboration links. The line thickness in the network map depicts the collaboration frequency between countries. The United States had a visibly dominant collaboration node with a strong collaboration network with almost all other countries on the map. China, which had the second-largest node and the second-highest number of publications, collaborated mainly with the US, Denmark, Australia, and Canada.

## 4 Recent Research Advances of Capsaicin on Human Cancer

Non-communicable diseases, including cancer, contribute significantly to morbidity and account for over 70% of untimely global mortality. The vast plurality of these deaths (over 80%) occurs in nations with a low or medium Human Development Index (58). Cancer is considered an increasing public health concern with devastating economic implications (59). With the aging and growing population, about 29 million cancer cases are expected by 2040 (60). Cancer treatment is currently a topic of high interest due to its severity, impact on quality of life, and burden on the healthcare system (61). Despite the advancement of medical science, the burden of cancer keeps rising rapidly and demands safe and more effective cancer prevention and treatment strategies capable of inhibiting or reversing cancer (32). The failure of most of the current treatment strategies has been linked to the fact that different forms of cancer are capable of acquiring mutations that make them resistant to treatment over time.

Lately, however, there has been growing attention to the potential of natural products, including dietary phytochemicals such as capsaicin, as safe and effective therapies for combating cancer (62, 63). This current research paradigm stems from the role of diet in 30% of cancers and the proposition that 35% of cancer can be prevented by diet and lifestyle changes (64, 65). Indeed, dietary phytochemicals have shown significant efficacy in ameliorating several levels of cancer development. Equally advantageous is the fact that these dietary phytoconstituents are readily available, relatively non-toxic, biocompatible, and cheap.

About 25% of global therapeutic drugs have been sourced primarily or otherwise from plants (66). For instance, anticancer agents such as vinblastine and paclitaxel were derived from *Catharanthus roseus* (L.) G. Don (syn. *Vinca rosea* L.) and *Taxus brevifolia* Nutt*.*, respectively (62). Thus, natural therapeutic agents are historically key contributors to drug discovery for various diseases, including cancer (67). Persuasive evidence from experimental and epidemiological studies has shown that some of these plant-derived therapeutic agents possess promising chemopreventive and chemotherapeutic properties (64). Capsaicin, an homovanillic acid derivative, is one of such dietary phytochemicals with the ability to ameliorate cancer at various levels. Various remedial effects and mechanisms of actions of capsaicin have been documented (2, 32, 61). This section considers various anticancer roles of capsaicin and the underlying mechanism in a more detailed perspective.

### 4.1 Capsaicin and Chemoprevention

Chemoprevention refers to the use of chemotherapeutic agents that hinders and halts the development of tumor before the onset of tumor cell invasion (68). Capsaicin has shown significant prospects as an effective chemopreventive agent, as discussed below.

#### 4.1.1 Antimutagenic Activity of Capsaicin

Arguably, the initial evidence of the anticancer role of capsaicin could be traced to earlier studies on the chemopreventive/anticarcinogenic activity. Capsaicin pre-treatment could suppress DNA binding of benzo (a) pyrene (a carcinogenic polycyclic aromatic hydrocarbon), thereby inhibiting lung carcinogenesis in mouse model (69). Several studies further corroborated this research finding with capsaicin showing protective effects against chemical carcinogens such as aflatoxin B1, 4-(methylnitrosamino)- 1-(3-pyridyl)- 1-butanone, vinyl carbamate and N-nitrosodimethylamine (70). It is noteworthy that a number of these hydrocarbons (specifically halogenated hydrocarbons) are metabolized by the phase drug-metabolizing enzyme CYP450 2E1, which catalyzes the activation to generate highly reactive genotoxic products. Interestingly, capsaicin has been found to inhibit several isoforms of CYP 450 enzymes, including CYP 2E1. As such, the chemoprotective role of capsaicin has been linked to its ability to modulate CYP enzymes (7). Furthermore, in a more recent study, capsaicin was shown to cause upstream activation of Ca^2+^/calmodulin (CaM)-dependent protein kinase (CaMK) and CCAAT/enhancer-binding protein β (C/EBPβ), which resulted in a concomitant inhibition of CYP1A1 mRNA (71). Hence, by inhibiting CYP enzymes expression and its upstream modulator, capsaicin is capable of acting as an anticarcinogenic agent.

#### 4.1.2 Anti-Oxidative Action of Capsaicin

Another plausible mechanism implicated in the chemopreventive action of capsaicin is its anti-oxidative effects. Capsaicin elicits a biphasic anticancer action, acting directly to scavenge free radicals and upregulating the expression of several antioxidant enzymes. Antiradical activity of pure capsaicin revealed high scavenging activity against 2,2′-azino-bis(3- ethylbenzothiazoline-6-sulphonic acid (ABTS) radical with IC_50_ value of 187.7 µM (72). Likewise, there was a positive correlation between the levels of capsaicin and its analogues and the antioxidant activity of peppers of the genus *Capsicum* (73). Capsaicin was also shown to protect against autoxidation and Fe^2+^ induced oxidation of linoleic acid (74). In addition, capsaicin inhibits reactive oxygen species (ROS) release and the subsequent mitochondrial membrane potential collapse, cytochrome c expression, chromosome condensation, and caspase-3 activation induced by oxidized low-density lipoprotein in human umbilical vein endothelial cells (75). This profound free radical scavenging activity gives credence to the ability of the compound to mitigate oxidative stress conditions, which have been implicated in cellular dysfunction vis-a-vis the development of cancer.

Aside from directly scavenging free radicals *in vitro*, capsaicin has also been found to increase the expression of antioxidant enzymes *in vivo* to modulate oxidative imbalance. Capsaicin pre-treatment in mice suppressed oxidative damage in mice testicles exposed to heat stress by modulating heat shock 70-kDa protein 1 (Hsp72), phospholipid hydroperoxide glutathione peroxidase (PGHPx), and manganese superoxide dismutase (MnSOD) mRNA expression (76). Hsp72 gene is upregulated in response to oxidative stress; however, the pre-exposure to capsaicin results in decreased Hsp72 levels (76). Likewise, the increased expression of MnSOD and PGHPx underscores the role of capsaicin in activating antioxidant enzyme expressions. A similar protective effect was demonstrated in cisplatin-induced nephrotoxicity in rats, where exposure to capsaicin decreased the levels of kidney malondialdehyde and ameliorate decreased levels of GSH and SOD activity (77).

**Figure 4:**
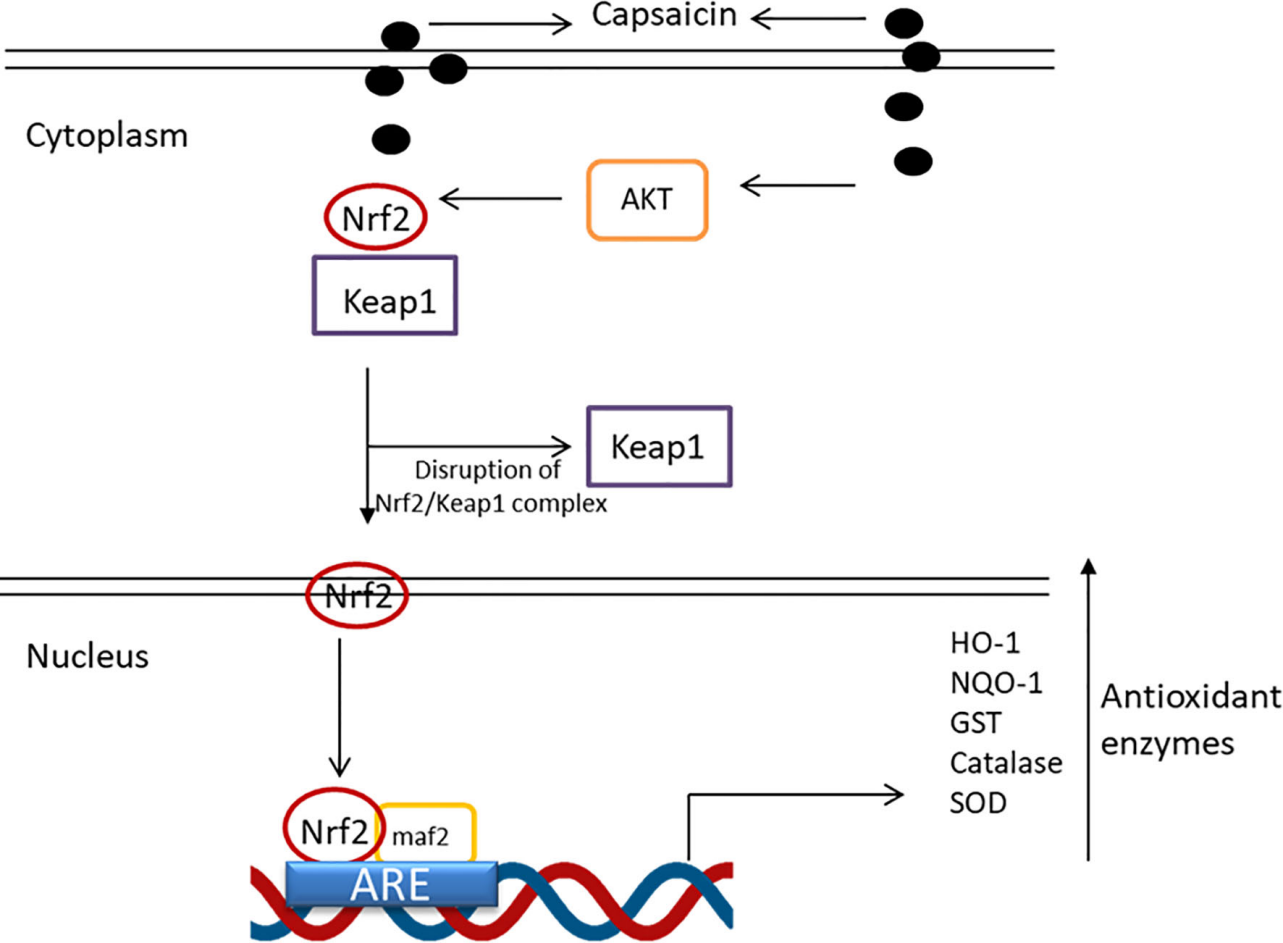
Antioxidant enzyme pathway regulation by capsaicin.

Additionally, capsaicin can act synergistically with other dietary phytochemicals causing an exponential beneficial cytoprotective effect (78, 79). For instance, dietary curcumin and capsaicin concurrent administration in high-fat diet-fed rats were shown to mitigate the testicular and hepatic antioxidant status by increasing GSH levels, glutathione transferase activity, and Cu-ZnSOD expression (79). The investigations by Joung et al. (80) provided further mechanistic insights into the antioxidant defense mechanism of capsaicin. The authors noted that capsaicin was capable of inducing a series of protein kinase phosphorylation events activating the antioxidant defense response in HepG2 cells. Capsaicin was shown to trigger the phosphorylation of Akt, activating the protein kinase leading to Nrf2 phosphorylation (80). The phosphorylation of Nrf2 results in disruption of NRF2/Keap 1 complex liberating the activated Nrf2 protein, which translocates to the nucleus forming a complex with maf2, which binds to the antioxidant response element in the promoter region of genes encoding the antioxidant enzyme heme-oxygenase-1 (**Figure 4**). HO-1 catalyzes the oxidative degradation of heme to liberate free heme, carbon monoxide and biliverdin. By degrading heme, HO-1 prevents oxidative damage by heme protein. Besides HO-1, Nrf2 activation have also been linked to the increased expression of other and drug-metabolizing enzymes such as NAD(P)H:quinone acceptor oxidoreductase (NQO) and antioxidant enzymes, including catalase (CAT), SOD, GPX and GST *via* the Nrf2/ARE pathway (81).

#### 4.1.3 Anti-Inflammatory Action of Capsaicin

Anti-inflammation is another mechanism implicated in the chemopreventive action of capsaicin. According to the national cancer institute, chronic inflammation has been named as a major risk factor in cancer. This is because, during chronic inflammation, notable damage to the DNA structure is observed, which can ultimately result in cancer. Such is the case observed during chronic inflammatory bowel disorders such as Crohn’s disease and ulcerative colitis, which leads to colon cancer. Sub-plantar injections of capsaicin were able to significantly inhibit paw-swelling in Wistar rats at a rate comparable to standard drug diclofenac (82). The anti-inflammatory effect of capsaicin was initially linked to capsaicin receptors known as transient receptor potential vanilloid sub-type1 (TRPV1). Vanilloid receptors have been implicated in tissue injury and inflammation; however, repeated application of capsaicin results in anti-inflammatory responses *via* these receptors. Recent studies have suggested that the anti-inflammatory action of capsaicin is independent of the TRPV1 receptor (83–85).

**Figure 5:**
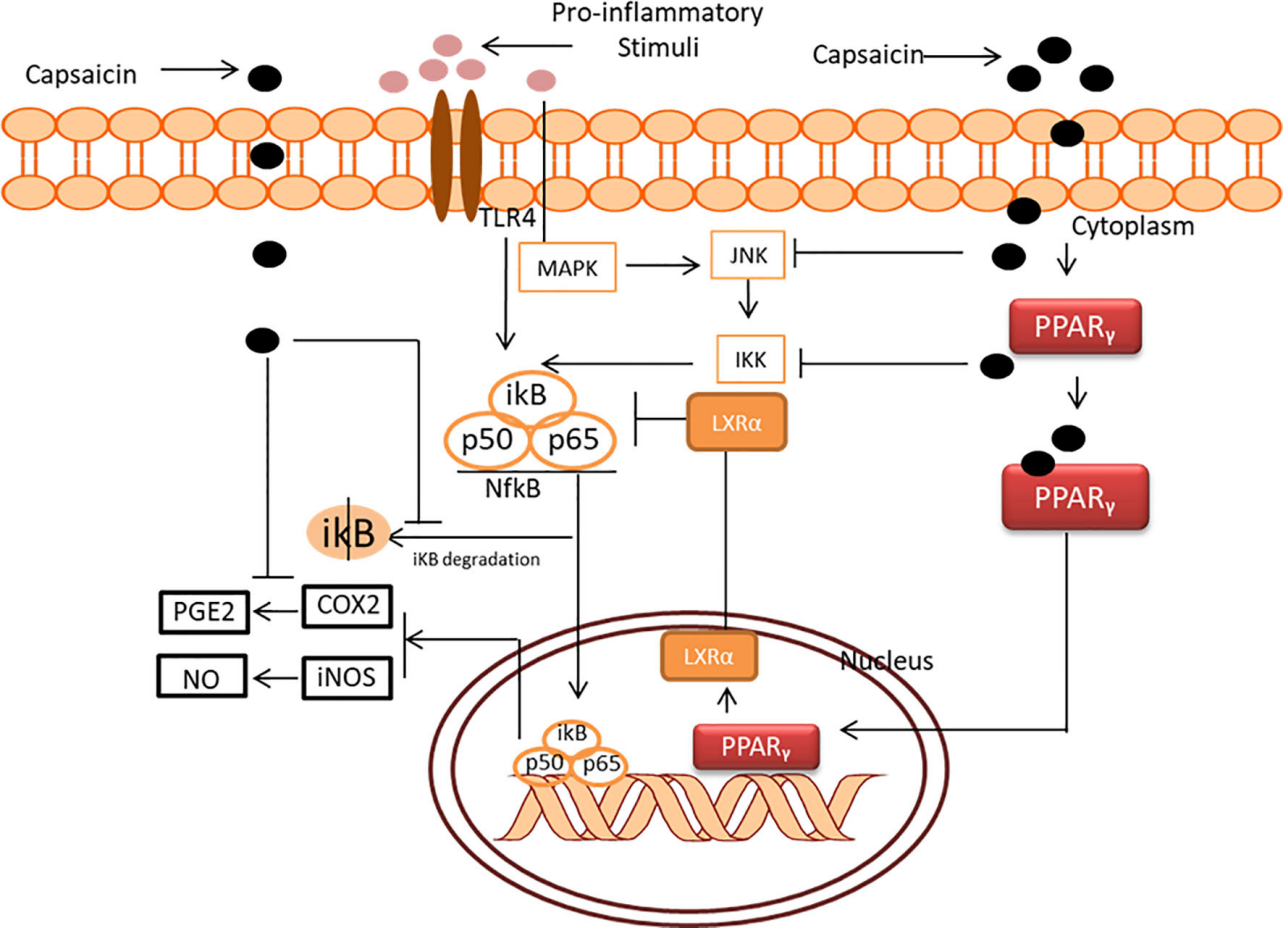
Anti-inflammatory mechanism of capsaicin.

Capsaicin mediates anti-inflammation by inhibiting lipopolysaccharide (LPS)-induced IL-1β, IL-6 and TNF-α production by increasing Liver X receptor α (LXRα) expression through peroxisome proliferator-activated receptor-gamma (PPARγ) pathway (85). The authors also observed that the activation of LXRα blocks NF-κB-mediated inflammatory gene expression and the inhibitory action of capsaicin on NF-κB expression was blocked by LXRα inactivation with siRNA. Likewise, capsaicin inhibits toll-like receptor-mediated salivary epithelial cells’ release of pro-inflammatory cytokines through the NF-kB signalling pathway (84). Kim et al. (83) reported the inhibition of NF-kB by capsaicin *via* a mechanism involving the degradation of ikB-α. The compound elicits COX-2 enzyme activity inhibition and downregulation of iNOS protein to ameliorate inflammation in LPS-stimulated murine peritoneal macrophages. Chen et al. (86) investigated the signal transduction mechanism implicated in the anti-inflammation action of capsaicin in RAW264.7 macrophages. Capsaicin inhibited LPS- and IFN-γ-mediated NO production, iNOS protein and mRNA expression, COX-2 expression and PGE2 production. In addition, capsaicin inhibits NF-κB, AP-1 activation and STAT1 activation, as well as other upstream protein kinases, including ERK, JNK and IKK. The inhibition of the upstream kinase is implicated in the apoptotic action of capsaicin, which is discussed in the subsequent session. The overall anti-inflammatory of capsaicin is summarized in **Figure 5**.

#### 4.1.4 Cell Cycle Regulation by Capsaicin

Cell progresses through the G0/G1, S and G2/M phases of the cell cycle during cell proliferation. This series of events is highly regulated by cyclin, cyclin-dependent kinase, and checkpoint kinases, including polo-like kinase, aurora kinase and CDK inhibitors which ensures that damaged/mutated cells do not proceed through in cell cycle (87). However, in cancer cells, deregulation in cell cycle regulations allows cell proliferation to occur. Over the years, dietary phytochemicals such as capsaicin have shown attractive cell cycle regulation activity, thereby halting cellular division of cancer cells (87). Upon sensitization by proliferative stimulus, cell progresses from the resting G0 phase to the growth phase (G1). A recent study observed that capsaicin mediates cell cycle arrest at the G1 phase in ORL-48 cells (88). Similarly, Qian et al. (89) reported G0/G1 cell cycle arrest in bladder cancer cells following capsaicin treatment. However, studies have also reported the inhibition of the cell cycle at the G2/M phase in human KB cancer cells and MCF7 breast cancer cells. The cellular arrest is usually achieved by modulation of cell cycle protein kinases. For instance, downregulation of CDK8 expression was involved in the G2/M phase arrest of breast cancer cells by capsaicin (90). In another study, the inhibition of CDK2, CDK4 and CDK6 were responsible for G0/G1 arrest (91). Likewise, the anti-tumor effect of capsaicin on human pharyngeal squamous carcinoma cells (FaDu) is associated with mitochondrial pathways, possibly by decreasing the expression of the regulators of cyclin B1 and D1, as well as cyclin-dependent protein kinases CDK-1, CDK-2 and CDK-4 mediating cell cycle arrest at G1/S phase (92).

Beyond the anti-CDK activity of capsaicin, capsaicin modulates upstream molecular events such as the p53 dependent pathways. Islam et al. (93) recently reported that tumour-associated NADH oxidase (tNOX) is a major target of capsaicin responsible for its effect on the cell cycle. The authors noted that modulating tNOX reduces NAD+ generation and inhibits SIRT1, causing c-myc and p53 activation, ultimately leading to the inhibition of cyclin/CDK complex at G1 checkpoint triggering cell cycle arrest. In bladder cancer, capsaicin treatment down-regulates tNOX and SIRT1 expression prolonging cell cycle progression among other effects (94). Capsaicin elicits anticancer effect *via* a p53 dependent pathway in human colon cancer cells (95). By suppressing p53/MDM2 interaction, capsaicin inhibited p53 degradation, allowing p53 to induce cell cycle arrest at the G0/G1 phase and apoptosis (95). In addition, *via* a mechanism that involves the vanilloid receptor TRPV1, capsaicin modulates the expression of p53, p21 and CDK2, initiating G0/G1 phase arrest in bladder cancer RT4 cells (96). Overall, by modulating critical signal transducers in the cell cycle, capsaicin can halt cancer proliferation in different cancer types to prevent the progression of cancer.

### 4.2 Cell Death Mechanism of Capsaicin

Apart from being a chemopreventive agent, capsaicin has shown cytotoxic effects. It has been reported to cause the induction of cell death in different cancer cells in *in vitro* and *in vivo* models.

#### 4.2.1 Apoptotic Cell Death by Capsaicin

Apoptosis is the primary mechanism *via* which capsaicin can induce cell death in cancer cells, including prostate cancer, pancreatic cancer, colorectal cancer, lung cancer, breast cancer, liver cancer, and skin cancer. Apoptosis is a programmed form of cell death characterized by morphological and biochemical events including membrane blebbing, cell shrinkage, nuclear and DNA fragmentation, chromatin condensation followed by engulfment of the dead cells by neighbouring cells (97). A different molecular mechanism capable of inducing apoptosis has been described following the treatment of cancer cells with capsaicin.

One of the major pro-apoptotic mechanisms of capsaicin is *via* the vanilloid receptors, primarily TRPV1, a non-selective calcium channel that has been functionally involved in cell death in a wide variety of cancer cells. In glioma cells, capsaicin treatment increased the expression of TRPV1, causing a concomitant influx of Ca^2+^ triggering apoptosis *via* the p38 signalling pathway (98). Similarly, in anaplastic thyroid cancer, the agonistic role of capsaicin led to the inhibition of the cell viability as a result of cell death *via* the intrinsic pathway of apoptosis (99). The mechanism also involved triggering Ca^2+^ influx into the cell cytoplasm, causing an imbalance in intracellular calcium homeostasis and a severe condition of mitochondria calcium overload (99). The disruption of the mitochondria calcium balance resulted in increased production of mitochondria reactive oxygen species, depolarization of mitochondria membrane potential, and opening of mitochondria membrane permeability pore (99). The latter effects result in the release of cytochrome C, triggering apoptosome assembly and the activation of caspase, leading to apoptotic cell death. Equally worth emphasizing is that the study also showed that in the presence of TRPV1 antagonist and calcium chelator, apoptosis underscores the role of the TRPV1 receptor pathway in capsaicin-induced apoptosis in cancer cells (99).

**Figure 6:**
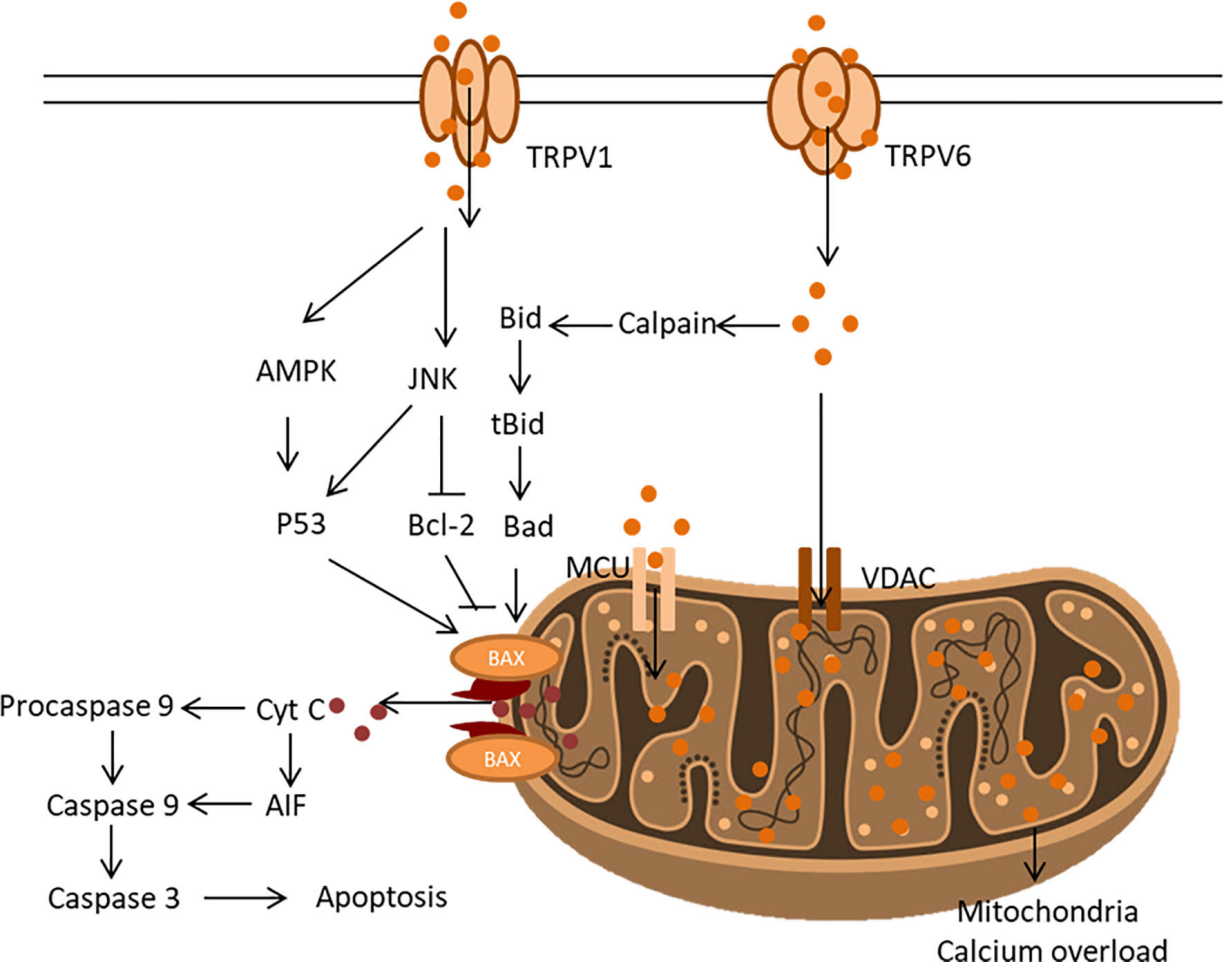
Intracellular signalling pathway implicated in the apoptotic action of capsaicin.

Amantini et al. (100) studied the role of TRPV1 in capsaicin-induced apoptosis in human urothelial cells. The authors noted that TRPV1 dependent apoptosis involved the activation of pro-apoptotic protein -ataxia-telangiectasia mutated (ATM), which is involved in Ser15, Ser20, and Ser392 phosphorylation in the DNA damage response pathway, and the activation of Fas/CD95 protein which mediates intrinsic and extrinsic apoptosis pathway. Likewise, TRPV1 activation following capsaicin treatment results in apoptosis induction in colorectal cancer *via* the calcineurin-NFAT2-p53 signaling pathway (101). Aside from TRPV1, another member of the TRPV family which has been implicated in the apoptotic action of capsaicin is TRPV6. Like TRPV1, TRPV6 is a calcium selective ion channel that regulates calcium homeostasis. In human small cell lung cancer, capsaicin displays potent antineoplastic activity by increasing TRPV6 expression, causing increased levels of intracellular calcium ions activating the calpain pathway to induce apoptosis (102). According to Chow et al. (103) TRPV6 mediated capsaicin-induced apoptosis activation in gastric cancer cells. It was observed that TRPV6 overexpression increased mitochondria permeability in the cells through the activation of Bax and p53 through C-jun N-terminal kinase (JNK) activation (**Figure 6**).

Perhaps, what makes capsaicin a particularly interesting anticancer agent is its ability to act on multiple anticancer targets? This feature is once again exemplified in its apoptotic mechanism, which has been found not to be limited to vanilloid receptor pathways (104). The studies by Bao et al. (105) revealed that by treating human osteosarcoma MG63 cells with capsaicin, apoptosis by induced *via* the TRPV1-dependent and independent pathways. Through the TRPV1 independent pathway, capsaicin-induced apoptosis by activating adenosine-5-monophosphate-activated protein kinase (AMPK), p53 and JNK. Findings by Kida et al. (106) showed that in the presence of TRPV1 antagonist, capsaicin stimulated intracellular calcium influx. Likewise, the binding of capsaicin to mitochondrial complex I and II in the electron transport chain disrupted the mitochondrial membrane potential and increasing mitochondrial membrane permeability (107). Zhang et al. (108) equally showed that capsaicin increases ROS levels and results in increased expression of pro-apoptotic Bcl-2 (Bax), downregulation of anti-apoptotic Bcl-2 and CytC release, causing cell death in pancreatic cancer cells *in vitro* and *in vivo*. Thus, *via* a mechanism independent of TRPV1, capsaicin can activate the extrinsic and intrinsic apoptosis pathways.

#### 4.2.2 Autophagy Mediated Cell Death by Capsaicin

Autophagy is a highly regulated process through which cytoplasmic components are delivered to the lysosome for degradation and later recycled to meet the metabolic needs of starving cells (68, 109). While it was initially thought of as a pro-survival mechanism, autophagy has been found to play a dichotomous role as a cell survival mechanism and cell death mechanism (68, 109). Although the role of capsaicin in autophagic death is yet to be fully understood, it appears to vary with different cancer cell types as it has been shown to inhibit or promote autophagy in different forms of cancer (110–112). By blocking the Pi3/Akt/mTOR signalling pathway, capsaicin increases levels of autophagic markers (LC3-II and Atg5), enhances p62 and Fap-1 degradation and increases caspase-3 activity to induce apoptosis in human nasopharyngeal carcinoma cells (112). Moreover, capsaicin acts through tNOX to induce autophagic apoptosis in oral and melanoma cancer cells (113, 114). Conversely, in U251 Glioma cells, capsaicin was shown to inhibit autophagy, and this inhibition resulted in increased apoptotic cell death (110). As such, in these cancer cells, autophagy appears to be a pro-survival mechanism and its inhibition by capsaicin results in cell death.

However, most studies have suggested that capsaicin is likely to induce autophagy in cancer cells in a manner that assists in cancer cell survival. For instance, in the study by Chu et al. (111), it was shown that capsaicin-induced autophagy, which served a tumour-promoting role in human melanoma cells and inhibition of autophagy using 3-MA enhanced capsaicin-induced cell death. Similarly, Chen et al. (115) increased stat3 dependent autophagy through the generation of ROS in human hepatoma (HepG2 cells) and inhibition of autophagy enhanced capsaicin-induced apoptosis. Further studies have shown that autophagy induction by capsaicin retards cell death by suppressing endoplasmic reticulum stress-mediated apoptosis *via* a pathway involving JNK, p38 and ERK (116, 117).

#### 4.4.3 Necrosis, Paraptosis, and Necroptosis Cell Death by Capsaicin

Although apoptosis is the main form of cell death described in capsaicin-treated cancer cells, other forms of cell death such as necrosis, paraptosis, and necroptosis have also been reported using *in vitro* and *in vivo* models (118, 119). Several factors, including cell type, dose and time of capsaicin exposure, can influence the type of cell death mediated by capsaicin (118). Capsaicin induces necrotic cell death in a time-dependent manner in 5637 and T24 BC cells and that autophagic inhibitor enhances the cytotoxic effects (120). Following the investigation of the effect of capsaicin-induced TRPV1 expression on cell proliferation in breast cancer, Wu et al. (121) observed that TRPV1 does not enhance cell proliferation and capsaicin was able to induce necrotic cell death in the MCF-7 cell, which was associated with increased expression of c-fos and RIP3. Ramírez-Barrantes et al. (122) equally established that TRPV1 expression mediates necrosis in HeLa cells. The authors observed that at high concentration (> 10 µM), capsaicin induces a slow but persistent increase in intracellular Ca^2+^, which leads to plasma membrane depolarization, mitochondrial dysfunction, and ultimately cell death by necrosis and apoptosis.

Another form of cell death has been described by Jambrina et al. (119), using capsaicin. The authors reported that activation of TRPV1 by capsaicin causes Ca^2+^ influx, which triggers a distinct program of mitochondrial dysfunction leading to paraptotic cell death, which does not fulfil the criteria for either apoptosis or necrosis. Huang et al. (123) also reported necroptosis (programmed necrosis) in oral squamous cell carcinoma cells treated with capsaicin. It is noteworthy that the role of paraptosis and necroptosis in the anticancer effects of capsaicin is yet to be fully elucidated. Further studies are thus required to carefully study this pathway of cell death in capsaicin-treated anticancer cells.

### 4.3 Capsaicin Intervention in Cancer Metastasis

Tumor cells, in certain cases, are capable of migrating through the lymphatic or blood systems to colonize distant sites through a process known as metastasis. This is a complex process implicated in 90% of cases of cancer mortalities, and it involves several alterations causing stimulation of angiogenesis, local invasion attachment, basement membrane disruption, matrix proteolysis, and stimulation of growth factors (68, 124). Capsaicin has been shown in the past to mitigate cancer metastasis due to the ability to modulate critical pathways involved in molecular alterations of cancer. For instance, Min et al. (125) described the inhibition of angiogenesis by capsaicin under *in vitro* and *in vivo* systems. Angiogenesis is the formation of new blood vessels to deliver nutrients and oxygen necessary for secondary tumor growth. By inhibiting angiogenesis, capsaicin can stall the growth of secondary tumours. In non-small lung cancer cells, capsaicin was able to restrain angiogenesis by dampening vascular endothelial growth factor (VEGF) expression *via* p53-SMAR1 auto-regulatory loop activation (126). Capsaicin is also able to inhibit tumor metastasis by inhibiting the matrix proteolysis pathway. Specifically, capsaicin has been shown to target matrix metalloproteinase 9 (MMP9), a protein responsible for extracellular matrix degradation and cytokine activation during tissue remodelling in metastatic cancer. The inhibition of MMP9 by capsaicin occurs *via* the suppression of AMPK-NF-κB, EGFR-mediated FAK/Akt, PKC/Raf/ERK, p38 MAPK, and AP-1 signaling pathway (127, 128). In addition, Shin et al. (129) outlined phosphatidylinositol 3-kinase/Akt/Rac1 signal pathway inhibition as the primary mechanism of cell migration in B16-F10 melanoma. In human papillary thyroid carcinoma BCPAP cells, capsaicin inhibits matrix protease MMP9 and MMP2 by activating the TRPV1 channel (130). Based on recent evidence (131), capsaicin might inhibit migration and invasion and metastasis of oesophageal squamous cell carcinoma (ESCC) *via* overexpression of claudin-3 (Cldn3) and inhibiting epithelial-mesenchymal transition (EMT). The anti-metastatic effect of capsaicin has been further validated using *in vivo* mouse prostate cancer model where it was demonstrated that capsaicin significantly reduced the metastatic burden (132).

### 4.4 Human Clinical Trials on Capsaicin

Within the last two decades, there have been several clinical reports on the use of capsaicin. However, most of the studies have mainly examined the analgesic activity of capsaicin. There have been few reports on the use of capsaicin in cancer patients; however, these studies have examined the pain relief function of capsaicin in addition to other treatment regimens (133–135). For instance, Adlea (ALRGX-4975) - an injectable preparation of capsaicin in phase II clinical trials in Morton’s neuroma patients, was effective in the treatment of chronic neuropathic pain (134). Likewise, Privitera and Anand (133) revealed that capsaicin 8% patch could promote the regeneration and restoration of skin nerve fibres in chemotherapy-induced peripheral neuropathy in addition to pain relief. These studies have suggested that capsaicin might serve as a suitable adjuvant to ameliorate pain and its associated complications in cancer treatment. However, it remains unclear whether capsaicin serves any antiproliferative function in cancer patients. Further clinical studies focused on its antiproliferative potential are therefore required.

### 4.5 Downsides and Recent Improvement Initiatives on the Effects of Capsaicin on Human Cancer

The last few decades have seen a significant increase in the number of research conducted on the anticancer effects of capsaicin, yet none of these research works has been met by clinical approval. This limitation stems from certain challenges with capsaicin as a drug. One of the limitations of capsaicin is its pro-carcinogenic effects. Although the number of anticancer studies of capsaicin far outweighs the carcinogenic activity, nonetheless, the risk associated with capsaicin carcinogenic effect is a major concern to researchers (136). Another major drawback of capsaicin is the lead-likeness property. Capsaicin has been shown to have high hydrophobicity, low binding affinity, and short half-life, which can affect the *in vivo* anticancer efficacy (31). More so, capsaicin has shown several unpleasant side effects, including stomach cramps, skin and gastric irritation, and burning sensation (137). Hence, we further discuss the recent studies carried out to improve the anticancer efficacy of capsaicin.

#### 4.5.1 Synthesis of Capsaicin and Its Analogs

The majority of capsaicin used in research have been purified from *Capsicum* plants, with varying levels of purity which has led to a disparity in some results obtained from biological assays (138). To circumvent these challenges, capsaicin has been synthesized artificially with a high level of purity and high yield. Furthermore, to bypass some side effects and limitations with capsaicin, different capsaicin analogs have been synthesized some of which have shown significant anticancer prospect. A typical capsaicin structure consists of an aromatic ring (region A), an amide group (region B), and a hydrophobic group (region C) (137). Modifications of capsaicin pharmacophore have focused mainly on the B and C region of the capsaicin structure to yield capsaicinoids such as capsiate, dehydrocapsaite, nordihydrocapsiate, which have only shown anticancer properties without any reported carcinogenic effects (137). In a study by Lewinska et al. (139), capsaicin epoxide was synthesized and found to be non-toxic to human dermal fibroblast cell lines and showed higher toxicity to cancer cell lines compared to capsaicin by inducing oxidative damage. Likewise, by modifying regions A and B of capsaicin, Pereira et al. (140) synthesized a capsaicin-like analogue which induced apoptotic cell death in cancer cells with a better pharmacokinetic profile than capsaicin and had no irritant effects on mice. These findings were corroborated by de-Sá-Júnior et al. (141), who synthesized RPF101, by modifying similar aromatic and amide substituent groups. The compound had a better pharmacokinetic profile than capsaicin and was reactive toward the cancer target. In addition, capsazepine − a TRPV1 antagonist, has also been synthesized and found to be a highly effective pleitropic antitumoral/anti-inflammatory agent in cancer cells and *in vivo* models (142). So far, the several capsaicin analogs have shown significant promise but require further *in vivo* and clinical validations.

#### 4.5.2 Targeted Delivery of Capsaicin

In an effort to enhance the bioavailability, improve the pharmacokinetics and half-life and reduce the side effects, different delivery vehicles, including inorganic carriers (metal nanoparticles and carbon sphere), polymeric carriers (micelle, dendrimer and polymersome), and lipid-based nanoparticles (liposomes, microencapsulation and solid-lipid nanoparticle) have been developed to perform site-directed delivery of capsaicin (143). In addition, excipient-free self-assembled capsaicin delivery systems have been designed with improved pharmacokinetic properties (144). Studies on the delivery of capsaicin for improved anticancer functions are summarized in **Table 1**.

**Table 1:** Recent examples of anticancer studies focusing on capsaicin delivery.

Delivery system	Experimental model	Cancer form	Effective dose	Main findings/activity	Conclusion	References
Capsaicin loaded albumin nanoparticles	*In vivo* rat model	Inflammation/cancer chemoprevention	50 mg/kg intraperitoneal administration	The nanocomposite showed concentration and time-dependent *in vitro* antioxidant activity. In *in vivo* rat model, the nano-encapsulated drug reduced TNF-α concentrations.	The albumin NPs are potential capsaicin carriers applicable in several conditions, including cancer and inflammation.	(145)
Capsaicin loaded solid-lipid nanoparticles	*In vitro*	Hepatocellular carcinoma	IC_50_ of 21.36 µg/ml	The nanoparticle-loaded drug showed anticancer property against HepG2 cells *in vitro*. The capsaicin loaded NPs were stable in circulation for up to 3 days.	The capsaicin loaded nanoparticles showed improved pharmacokinetic property as well as enhanced anticancer property.	(146)
Capsaicin loaded folic acid conjugated lipid nanoparticles (CFLN)	*In vitro*	Ovarian cancer	>50µg/ml	CFLN had significant cell apoptosis (39%) compared to capsaicin-loaded lipid NPs (21%) and pure capsaicin (11%).	The drug nanosystem had remarkable anticancer effects compared to pure capsaicin due to improved pharmacokinetics and active cancer cell targeting	(147)
NIR-triggered plasmonic nanodots capped mesoporous silica nanoparticles loaded with capsaicin	*In vitro*	Thyroid cancer	6 – 25 µM	The drug-loaded NPs exhibited extraordinary *in vitro* antitumor activity against thyroid cancer cell lines by inducing apoptosis. In addition, anti-metastatic activity was also observed.	The capsaicin-loaded NPs are potential candidates for cancer therapy.	(148)
Capsaicin-loaded trimethyl chitosan nanoparticles	*In vitro*	Hepatocellular carcinoma	50 -100 µ M	The capsaicin-loaded nanoparticles significantly improved the anticancer activity of capsaicin by inducing apoptosis compared to free capsaicin.	The drug delivery system improved the chemotherapeutic efficacy of capsaicin.	(149)
Capsaicin loaded hyaluronic acid nanoparticles	*In vitro* and *in vivo*	Lung cancer	20-50 µM *in vitro* concentration and 20 mg/kg, i.v. administration in rat	The loaded NPs significantly suppressed cancer cell viability compared to free capsaicin. The drug-loaded nanosystem also significantly reduced tumor volume in lung carcinoma in a rat model.	Significant anticancer effects both *in vitro* and *in vivo* were observed using the drug delivery system.	(150)
Capsaicin loaded nano-liposomes	*In vitro*	Breast cancer and pancreatic cancer	13-100 µM	The capsaicin-loaded nano-liposomes showed significant improvement in anticancer activity against cancer cells such as breast cancer cells and pancreatic cancer cell lines.	The capsaicin loaded nanoliposomes enhanced anticancer activity, improved pharmacokinetic property, and capsaicin selectivity compared to free capsaicin.	(151)
Capsaicin-in-cyclodextrin inclusion complexes loaded into pegylated liposomes	*In vitro*	Chemopreventive and cytotoxic effect on breast cancer cell	–	Liposome-based capsaicin significantly reduced IL-8 production by the MDA-MB-231 and A549 cancer cell lines after treatment.	Liposomes based delivery of capsaicin improved chemopreventive function.	(152)
Capsaicin-BODIPY self-assembly	*In vitro* and *In vivo* nude mice model	Prostate cancer	20-100 µM *in vitro* dose, 18 µg/kg bodyweight intraperitoneal injection in nude mice	Capsaicin covalently attached to BODIPY self-assemble in an aqueous solution and show improved delivery to tumor tissue. The capsaicin-based drug showed a 2-fold increase in antitumor activity in *in vivo* prostate cancer compared to free capsaicin.	The nanosystem ensured an active cancer target and showed significant biomedical potential.	(153)

Delivery systems such as nanoparticles offer the advantage of increasing the retention time in the blood system, thereby allowing the drug to achieve maximum efficacy before being cleared from the body. Likewise, liposomes and micro-emulsion-based drugs have been known to significantly improve oral bioavailability and reduce the irritation of drugs (154). In addition, these delivery systems can be surfaced-modified to perform site-directed/cell-specific drug delivery, thereby ensuring increased cell death of cancer cells while sparing non-selective normal cells (155). Furthermore, owing to its antioxidant potential, capsaicin has been applied asa bioreduction and capping agent to synthesize biocompatible silver nanoparticles and can be used in cancer theranostics (156).

#### 4.5.3 Drug Synergism and Capsaicin-Combination Therapy

The current generation of cancer therapeutics has good initial efficacy but often develops resistance within months of treatment. One way of combating this problem is through drug-drug combination and combination of chemotherapeutic treatment with other anticancer therapies such as radiotherapy and photothermal therapy. In the same vein, capsaicin has been combined with other anticancer therapies for more pronounced anticancer effects (**Table 2**). In most cases, the combination of capsaicin with other chemotherapeutic drugs has shown a significant synergistic effect. Recent studies have shown that capsaicin may also serve additional benefits as an adjunct to current chemotherapeutic drugs. The unique TRPV1 dependent cell death mechanism of capsaicin together with other cell death pathways by chemotherapeutic drugs ensures complete clearance of the cancer cells and makes it less likely for cancer cells to develop resistance.

**Table 2:** Examples of studies on capsaicin combination therapy for improved anticancer property.

Combination therapy	Experimental model	Cancer form	Effective dose	Main findings	Conclusion	References
Folic acid-functionalized co-therapy of capsaicin (Cap) and gefitinib (Gnb) nanoparticles	*In vitro* and *in vivo* (Wistar rats)	Lung cancer	*In vitro* dose 20-100 µM (Gnb and Cap combined in 2:1). *In vivo* therapy consists of 20 mg/kg of Gnb and 10 mg/kg capsaicin applied intraperitoneal.	Co-administration of gefitinib and capsaicin NPs displayed significant targeting potential and reduced tumor volume while restoring the biochemical parameters. Significant downregulation was observed for anti-apoptotic proteins (MMP-9) and up-regulation of pro-apoptotic proteins (caspase-3, caspase-9 and MMP-9) with co-therapy of gefitinib and capsaicin NPs, when compared with individual therapy through Gnb/Cap.	Co-administration of gefitinib and capsaicin is highly effective for the treatment of lung carcinoma.	(157)
Capsaicin-5-Flurouracil (5-FU) drug combination	*In vitro* and *in vivo* nude mice	Cholangiocarcinoma	*In vivo* dosage of 60 mg/kg 5FU and 150 mg/kg Capsaicin. 60 µM Cap and 40 µM 5FU were highly cytotoxic to CCA QBC939 cell line.	The combination of capsaicin with 5-FU was synergistic, with a combination index (CI) < 1, and the combined treatment also suppressed tumor growth in the cholangiocarcinoma xenograft to a greater extent than 5-FU alone. Capsaicin inhibits 5-FU-induced autophagy by activating the phosphoinositide 3-kinase (PI3K)/protein kinase B (AKT)/mammalian target of rapamycin (mTOR) pathway in cholangiocarcinoma cells.	Capsaicin may be a useful adjunct therapy to improve chemosensitivity in cholangiocarcinoma.	(158)
Brassinin combined with capsaicin	*In vitro*	Prostate cancer	>100 µM brassinin and > 75 µM Cap	The combination significantly increased the cytotoxicity as compared to the monotherapy alone. Furthermore, proliferation, apoptosis, mitochondrial membrane potential, and colony formation were significantly inhibited, and anti-apoptotic-, proliferative-, and metastatic-related proteins were inhibited in the combination. Likewise, constitutive MMP-9/2 expression and their enzymatic activity, as well as cell migration and tumor cell invasion in PC-3 cells were inhibited in the combination group.	Brassinin in combination with capsaicin exerts synergistic anticancer effects in prostate carcinoma.	(159)
Co-delivery of Paclitaxel by a capsaicin prodrug micelle	*In vitro* and *in vivo* mice model	Breast cancer	0.1-10 µg/ml *in vitro* administration on cells. > 10 mg/kg body weight intravenous administration.	Polymeric micelles containing capsaicin delivered in combination with PTX achieved 62.3% apoptotic tissue, compared to 45.4% apoptotic tissue when PTX was administered alone. *In vivo* antitumor activity of PTX/CAP-loaded micelles was superior to that of the single independent treatments in mice.	The polymeric prodrug micelles are a promising nanosystem for achieving synergistic antitumor efficacy of chemotherapy drugs paclitaxel and capsaicin.	(160)
Capsaicin combined with cisplatin	*In vitro* and *in vivo* mice model	Osteosarcoma	100 µM Cap and 16.7 µM cisplatin *in vitro* concentration. Oral galvage consisting of 20 mg/kg bodyweight capsaicin and 4 mg/kg cisplatin	The combination of capsaicin and cisplatin had significant effects on apoptosis induction, cell cycle arrest and cell invasion inhibition in osteosarcoma cells compared with the individual-treatment groups and the control group. The co-treatment of capsaicin and cisplatin-induced pro-survival autophagy in OS cells by targeting reactive oxygen species (ROS)/JNK and p-AKT/mTOR signaling pathways and inhibited tumor growth in an osteosarcoma xenograft model.	Combination of capsaicin and cisplatin has strong inhibitory effects on osteosarcoma cells.	(161)
Genistein in combination with capsaicin	*In vitro* and *in vivo* rat model	Breast cancer	Topical application of 25 µmol/L genistein and 25 µmol/L Cap in mice. 50 µmol/L genistein and 50 µmol/L Cap *in vitro* administration in cancer cells.	*In vitro* MCF-7 breast cancer cells, genistein and capsaicin exhibited a synergistic anticancer effect by inducing apoptosis. Genistein in combination with capsaicin inhibits COX-2 expression by a pathway involving AMPK activation.	Genistein in combination with capsaicin exerts anti-inflammatory and anticarcinogenic properties.	(162)
Capsaicin and docetaxel combination	*In vitro* and *in vivo* mice model	Prostate cancer	20 µM docetaxel + 40 µM Cap *in vitro* administration. 2 mg/kg Cap and 10 mg/kg docetaxel *in vivo* treatment in mice.	Co-treatment with docetaxel and capsaicin notably decreased Akt and its downstream targets mTOR and S6 phosphorylation. The combined treatment also increased the phosphorylation of AMP-activated kinase (AMPK) and the phosphorylation of its substrate acetyl CoA carboxylase. *In vivo* experiments confirmed the synergistic effects of docetaxel and capsaicin in reducing the tumor growth of PC3 cells.	Combination of docetaxel and capsaicin represents a therapeutically relevant approach for the treatment of prostate cancer.	(163)
Capsaicin and camptothecin	*In vitro*	Lung cancer	>10 µM concentration each of Cap and camptothecin.	Human small cell lung cancers (SCLC) cells treated with 10 μm capsaicin and 1 μm camptothecin show increased calpain activity relative to each drug alone. Combination of Cap and camptothecin increases susceptibility of lung cancer cells to apoptosis. The synergistic activity of capsaicin and camptothecin is mediated by the elevation of intracellular calcium and the calpain pathway.	Combination of camptothecin and capsaicin has the potential to be a feasible strategy for therapy and management of human SCLCs.	(137)
Curcumin and capsaicin	*In vitro* and *in vivo* mouse model	Liver cancer	10 – 27 µmol/mL capsaicin and curcumin combination were cytotoxic to cells *in vitro*. >5 mg/kg co-administration of Cap and curcumin reduced tumor volume *in vivo*.	Curcumin-capsaicin functionalized with glycyrrhetinic acid and galactose liposomes (CAPS-CUR/GA&Gal-Lip) effectively inhibited the expression of P-glycoprotein (P-gp) and Vimentin in HSCs+HepG2 (human hepatoma cell line) cocultured model *in vitro*. CAPS-CUR/GA&Gal-Lip exhibited lesser extracellular matrix (ECM) deposition, lesser tumor angiogenesis, and superior antitumor effect compared with the no- and/or Gal-modified Lip, which was attributed to the simultaneous blocking of the activation of HSCs and inhibition of the metastasis of tumor cells.	Co-delivery of Curcumin and capsaicin by Dual-Targeting Liposomes for Inhibition of aHSC-Induced Drug Resistance and Metastasis.	(164)
Capsaicin and sorafenib	*In vitro* and *in vivo* mouse model	Hepatocellular carcinoma	Sorafenib at 0-30 µmol/L in the presence of 50-100 µmol/L Cap inhibits liver cells. 50 mg/kg sorafenib and 200 µmol/L Cap inhibits tumor volume *In vivo*.	Combining capsaicin and sorafenib significantly enhanced the suppression of cell proliferation, achieving a high-level synergistic effect (inhibition rates over 50%) and promoting hepatocellular carcinoma (HCC) cell apoptosis. In nude mice with PLC/PRF/5 xenografts, combined administration of capsaicin and sorafenib significantly enhanced the suppression of tumor growth without apparent gross toxicity compared to either agent alone. Mechanistically, capsaicin (10–200 μmol/L) dose-dependently increased the levels of phosphorylated ERK (p-ERK) in PLC/PRF/5 cells, thus leading to enhanced sorafenib sensitivity and a synergistic suppression on the tumor cells.	Capsaicin-increased phosphorylation of ERK contributes to the enhanced antitumor activity of sorafenib, and capsaicin may be useful in improving the efficacy of sorafenib for the treatment of HCC.	(165)
Resveratrol and capsaicin combination with radiotherapy	*In vitro* and *in vivo* mouse model	Pancreatic adenocarcinoma	50 mg/kg Resveratrol and 5 mg/kg Cap with 2Gy irradiation of mice xenograft.	Combination of resveratrol and capsaicin radiosensitized tumor cells, but RT did not increase BFC combination toxicity in radioresistant tumor cells. Resveratrol and capsaicin addition to RT increased ROS production and led to significant tumor volume reduction in xenografted mouse preclinical model. The combination of resveratrol and capsaicin inhibited RT-induced DNA damage by keeping cells in the cell cycle, provoking exacerbated intrinsic apoptosis.	Resveratrol and capsaicin radiosensitize pancreatic adenocarcinoma towards cell death.	(166)
Sorafenib and capsaicin	*In vitro* and *in vivo* mouse model	Hepatocellular carcinoma	2.5 mg/kg bodyweight capsaicin administration in mouse. 40 µM Cap synergises with sorafenib in *in vitro* liver cells.	The combination of the two drugs had a much stronger inhibitory effect on both HepG2 and Huh-7 human HCC cells growth than either drug alone. The combination of capsaicin and sorafenib induces AMPK activation and Acetyl CoA carboxylase phosphorylation in HCC cells. *In vivo* experiments further showed that the antitumor effect of sorafenib was enhanced by its combination with 2.5 mg/Kg of capsaicin.	Combined treatment with capsaicin and sorafenib might improve sorafenib sensitivity, and therefore represents a promising and attractive strategy for the treatment of hepatocellular carcinoma cells.	(167)
Capsaicin and 3,3′-Diindolylmethane (DIM)	*In vitro*	Colorectal cancer	50 µM Cap and 12 µM DIM	Synergistic induction of apoptosis and inhibition of cell proliferation was observed in human colorectal cancer cells treated with the combination of capsaicin and DIM. The two compounds activated transcriptional activity of NF-κB and p53 synergistically.	Capsaicin and DIM work synergistically to inhibit cell proliferation and induce apoptosis in colorectal cancer.	(168)
Capsaicin in combination with doxorubicin	*In vitro*	Multiple cancer cells	>20 µM Cap potentiates the *in vitro* effect of Dox	Capsaicin synergistically enhanced the cytotoxicity of doxorubicin in Caco-2 and CEM/ADR 5000 cells. Capsaicin increased the intracellular accumulation of the fluorescent P-glycoprotein (P-gp) substrates rhodamine and calcein and inhibited their efflux from the MDR cell lines.	Capsaicin and piperine can overcome Multidrug resistance in cancer cells to Doxorubicin.	(169)
Capsaicin and pirarubicin	*In vitro*	Bladder cancer	200 nM pirarubicin combined with 150 µM Cap.	The activation of TRPV1 by capsaicin was shown to induce growth inhibition of 5637 cells in which TRPV1 was highly expressed. Activation of TRPV1 also enhanced the antiproliferative effects of pirarubicin using an MTT assay and cell cycle analysis.	Activation of TRPV1 by capsaicin enhanced the therapeutic efficacy of traditional chemotherapeutic drugs to treat bladder cancer.	(170)
Compostable polymeric nanoparticles (PPNPs) co-delivery of capsaicin (CAPS) and biotin (BT)	*In vitro*	Human gastric carcinoma	≥5 µM	Human gastric carcinoma cell lines, such as SGC-791 and NCI-N87, were induced to apoptosis *in vitro* by BT/CAPS@PPNPs.	BT/CAPS@PPNPs could be used as a new method to increase the efficacy of gastric therapeutics.	(171)
Capsaicin + radiotherapy (RT)	*In vitro* and *in vivo* nude mice	Prostate cancer	1-10 µM Cap and 1-8 Gy RT on pancreatic cells. Animals were treated with 5 mg/kg/d Cap with 6 Gy RT.	Capsaicin reduced colony formation rates and radio-sensitized human PCa cells (Sensitizer enhancement ratio = 1.3), which corresponded to the suppression of NFκB, independent of TRP-V1 receptor. *In vivo*, oral administration of capsaicin with RT resulted in a ‘greater than additive’ growth delay and reduction in the tumor growth rate greater than capsaicin (P < 0.001) or RT (P < 0.03) alone. Immunohistochemical analysis revealed a reduction in proliferation and NFκB expression, and an increase in DNA damage.	Capsaicin acts as a radio-sensitizing agent for prostate cancer through the inhibition of NFκB signalling.	(172)
Capsaicin and erlotinib	*In vitro*	Lung cancer	Cap (25 and 50 µM) and erlotinib (5 µM)	Capsaicin synergistically enhanced the cytotoxicity and cell growth inhibition of erlotinib in NSCLC cells, which were associated with the downregulation of ERCC1 expression and inactivation of AKT in A549 and H1975 cells.	Capsaicin with erlotinib is highly promising for lung cancer treatment.	(173)

## 5 Conclusion and future Perspectives

Evidence from this review highlighted the research trend and pattern (e.g., top authors, journals, and publications). It revealed an in-depth insight into the potential of capsaicin for managing human cancer. The last two decades have witnessed an increasing rate (an average of ca. 18% growth yearly) in the number of publications (dominated by research articles at 93%) following the renewed interest in capsaicin research. The research outputs have been majorly (ca. 42% of 3753 publications) produced by the United States, China, and Japan, which also had a visibly dominant collaboration node and network with most of the other countries identified in this review. Despite the evident productive collaboration, the inadequate representation of countries from the developing world remains a concern that needs to be addressed for significant success in exploring the potential of capsaicin for mitigating human cancer. The importance of concerted effort toward developing research collaboration between developed and developing countries cannot be over-emphasised. Based on the assessed eligible literature, the four keyword clusters generated and designated as thematic domains for capsaicin research included anti-cancer/pharmacokinetics, cytotoxicity, and *in vivo* neurological and pain research studies. The top-20 publications were distributed across multiple science-based subjects such as neurosciences, pharmacology, biochemistry, physiology, chemistry, cell biology, food science and technology, thereby suggesting the multidisciplinary approach currently being explored for capsaicin as potential therapeutics for several health conditions. In relation to the top-20 cited publications, the anti-cancer/pharmacokinetics/pharmacodynamics of capsaicin was the most active thematic domain in the last two decades.

The potential of capsaicin for mitigating cancer has been mainly explored for its chemopreventive effects and mechanisms involved in cell death as well as intervention in cancer metastasis. The chemopreventive effect of capsaicin is related to its ability to exert diverse biological effects, including anti-mutagenic, antioxidant and anti-inflammatory activities as well as cell cycle regulation. Furthermore, capsaicin demonstrated cytotoxic effects, often facilitated by the induction of cell death in different cancer cells under *in vitro* and *in vivo* models. Overall, capsaicin has shown effectiveness against various human malignancies in recent years. Although there is an increasing focus on assessing the clinical effects of capsaicin, especially the analgesic activity, the anticancer efficacy is currently limited. This has been attributed to the pro-carcinogenic effect, high hydrophobicity, low binding affinity and short half-life of capsaicin. Hence, more research efforts geared at mitigating these limitations remain pertinent. Some of the currently applied approaches entail synthesising natural and synthetic analogs, precise targeted delivery, drug synergism, and combination therapy for capsaicin. The potential of combination therapy for improved anticancer properties, especially for lung and prostate cancer, has demonstrated some promising results, which indicates the therapeutic value of capsaicin. Studies are required to identify capsaicin analogs with long-acting and greater anticancer effects. It is envisaged that promising results from these ongoing approaches (that address the existing limitations) will surely feed into future clinical studies on the anti-proliferative potential of capsaicin.

## Data Availability Statement

The original contributions presented in the study are included in the article/**Supplementary Material**. Further inquiries can be directed to the corresponding authors.

## Author Contributions

All authors listed have made a substantial, direct, and intellectual contribution to the work, and approved it for publication.

## Funding

TA acknowledges post-doctoral fellowship from the North-West University, South Africa. Support in form of the APC from the Faculty of Natural and Agricultural Sciences, North-West University is sincerely appreciated.

## Conflict of Interest

The authors declare that the research was conducted in the absence of any commercial or financial relationships that could be construed as a potential conflict of interest.

## Publisher’s Note

All claims expressed in this article are solely those of the authors and do not necessarily represent those of their affiliated organizations, or those of the publisher, the editors and the reviewers. Any product that may be evaluated in this article, or claim that may be made by its manufacturer, is not guaranteed or endorsed by the publisher.

## Supplementary Material

Supplementary Table 1 Main information on global capsaicin-related research from 2001 to 2021.

Supplementary Table 2 Top 20 author keywords (DE) and keywords-plus (ID) associated with capsaicin articles from 2001 to 2021.

Supplementary Table 3 Top 20 journal sources (of the 3753 articles) for capsaicin research from 2001 to 2021.

Supplementary Table 4 Leading authors in capsaicin research from 2001 to 2021 and their citation analysis.

Supplementary Table 5 Top 20 most cited research articles on capsaicin from 2001 to 2021 based on total citations (TC) and total citation per year (TC/Year).

Supplementary Table 6 Twenty top leading and most cited countries of capsaicin publications from 2001 to 202.

Supplementary Figure 1 Chemical structure of capsaicin.

Supplementary Figure 2 Capsaicin biosynthetic pathways (30, 174). PAL-phenylalanine ammonia lyase, CIAH-cinnamic acid 4-hydroxylase, COAH-coumaric acid 3- hydroxylase, CAMT-caffeic acid O-methyltransferase, PAT-putative aminotransferase, BCAT- branched chain amino acid transferase, IVD- isovalerate deshidrogenase, FAS-fatty acid synthase complex, KAS-β-ketoacyl-[acyl-carrier-protein] (ACP) synthase, ACL-acyl carrier protein, TATA- acyl-ACP thioesterase, ACS-acyl-CoA synthase, CS-capsaicin synthase.

Supplementary Figure 3 Flow chart of the bibliometric study of capsaicin showing the selection and inclusion criteria from Web of Science (WoS) and Scopus databases.

Supplementary Figure 4 Twenty most productive authors in capsaicin research (2001–2021).

Supplementary Figure 5 Network visualization map showing country collaborations on capsaicin research. The node represents countries. Node diameter signifies the collaboration strength of a country with other countries. Lines depict collaboration pathways between countries. Korea (North Korea and South Korea).
