# Supplementary material for: Capsaicin: A Two-Decade Systematic Review of Global Research Output and Recent Advances Against Human Cancer
Source: Front Oncol. 2022 Jul 13;12:908487. doi: 10.3389/fonc.2022.908487 (PMC9326111; doi:10.3389/fonc.2022.908487)
Supplement: Supplementary Table 1 — Main information on global capsaicin-related research from 2001 to 2021. [file Table_1.docx]

| **Data information** | **Results** |
| --- | --- |
| Time span | 2001:2021 |
| Sources (Journals, Books, etc.) | 1385 |
| Documents | 3753 |
| Average years from publications | 10.4 |
| Average citations per document | 24.58 |
| Average citations per year per doc | 2.022 |
| References | 94535 |
| **Document content** |  |
| Keywords Plus (ID) | 12586 |
| Author's Keywords (DE) | 6920 |
| **Authors** |  |
| Authors | 10113 |
| Author Appearances | 18716 |
| Authors of single-authored documents | 117 |
| Authors of multi-authored documents | 9996 |
| **Authors collaborations** |  |
| Single-authored documents | 155 |
| Documents per Author | 0.371 |
| Authors per Document | 2.69 |
| Co-Authors per Documents | 4.99 |
| Collaboration Index | 2.78 |
