# Supplementary material for: Capsaicin: A Two-Decade Systematic Review of Global Research Output and Recent Advances Against Human Cancer
Source: Front Oncol. 2022 Jul 13;12:908487. doi: 10.3389/fonc.2022.908487 (PMC9326111; doi:10.3389/fonc.2022.908487)
Supplement: Supplementary Table 2 — Top 20 author keywords (DE) and keywords-plus (ID) associated with capsaicin articles from 2001 to 2021. [file Table_2.docx]

| Rank | Authors' keywords | Articles | % of 3753 | Keyword-plus | Articles | % of 3753 |
| --- | --- | --- | --- | --- | --- | --- |
| 1 | Capsaicin | 2026 | 53.98 | Capsaicin | 1346 | 35.86 |
| 2 | TRPV1 | 250 | 6.66 | Male | 589 | 15.69 |
| 3 | Pain | 221 | 5.89 | Human | 398 | 10.60 |
| 4 | Apoptosis | 131 | 3.49 | Pain | 398 | 10.60 |
| 5 | Neuropathic pain | 109 | 2.90 | Controlled study | 383 | 10.21 |
| 6 | Inflammation | 84 | 2.24 | Rat | 380 | 10.13 |
| 7 | Nociception | 82 | 2.18 | Rats | 359 | 9.57 |
| 8 | Hyperalgesia | 80 | 2.13 | Female | 337 | 8.98 |
| 9 | Rat | 73 | 1.95 | Receptor | 317 | 8.45 |
| 10 | Substance P | 69 | 1.84 | Activation | 311 | 8.29 |
| 11 | Vanilloid receptor | 49 | 1.31 | Humans | 306 | 8.15 |
| 12 | Nuerogenic inflammation | 44 | 1.17 | Non-human | 299 | 7.97 |
| 13 | Calcitonin gene-related peptide | 42 | 1.12 | Neurons | 278 | 7.41 |
| 14 | CGRP | 42 | 1.12 | Animals | 269 | 7.17 |
| 15 | Cough | 42 | 1.12 | Hyperalgesia | 260 | 6.93 |
| 16 | Dihydrocapsaicin | 42 | 1.12 | Metabolism | 254 | 6.77 |
| 17 | Allodynia | 37 | 0.99 | Expression | 252 | 6.71 |
| 18 | Capsaicinoids | 36 | 0.96 | Adult | 243 | 6.47 |
| 19 | Oxidative stress | 35 | 0.93 | Mechanisms | 239 | 6.37 |
| 20 | Obesity | 33 | 0.88 | Substance P | 227 | 6.05 |
