# Supplementary material for: Capsaicin: A Two-Decade Systematic Review of Global Research Output and Recent Advances Against Human Cancer
Source: Front Oncol. 2022 Jul 13;12:908487. doi: 10.3389/fonc.2022.908487 (PMC9326111; doi:10.3389/fonc.2022.908487)
Supplement: Supplementary Table 3 — Top 20 journal sources (of the 3753 articles) for capsaicin research from 2001 to 2021. [file Table_3.docx]

| Journal | Publisher | Articles |
| --- | --- | --- |
| Pain | Lippincott Williams & Wilkins | 363 (10%) |
| European Journal of Pharmacology | Elsevier | 78 (2%) |
| Neuroscience Letters | Elsevier | 63 (2%) |
| Neuroscience | Elsevier | 56 (1%) |
| Brain Research | Elsevier | 55 (1%) |
| Journal of Pharmacology and Experimental Therapeutics | American Society for Pharmacology and Experimental Therapeutics | 39 (1%) |
| British Journal of Pharmacology | Wiley Blackwell | 38 (1%) |
| European Journal of Pain | Wiley Blackwell | 35 (1%) |
| Journal Of Agricultural and Food Chemistry | ACS | 35 (1%) |
| PLOS One | PLOS | 35 (1%) |
| Molecules | MDPI | 35 (1%) |
| Journal of Pain | Elsevier | 33 (1%) |
| Journal of Neurophysiology | American Physiological Society | 27 (1%) |
| Life Sciences | Elsevier | 26 (1%) |
| Scientific Reports | Nature | 25 (1%) |
| Molecular Pain | SAGE | 22 (1%) |
| Experimental Brain Research | Springer | 21 (1%) |
| Clinical Journal of Pain | Lippincott Williams & Wilkins | 20 (1%) |
| Journal of Neuroscience | Society for Neuroscience | 19 (1%) |
| Biochemical and Biophysical Research Communications | Elsevier | 19 (1%) |
