# Supplementary material for: Capsaicin: A Two-Decade Systematic Review of Global Research Output and Recent Advances Against Human Cancer
Source: Front Oncol. 2022 Jul 13;12:908487. doi: 10.3389/fonc.2022.908487 (PMC9326111; doi:10.3389/fonc.2022.908487)
Supplement: Supplementary Table 4 — Leading authors in capsaicin research from 2001 to 2021 and their citation analysis. [file Table_4.docx]

| Authors | H-index | g-index | m-index | TC | Articles | PY start |
| --- | --- | --- | --- | --- | --- | --- |
| Lee J | 20 | 28 | 1.0526316 | 901 | 46 | 2004 |
| Lee S | 21 | 41 | 0.9545455 | 1763 | 45 | 2001 |
| Wang Y | 17 | 25 | 0.7727273 | 861 | 43 | 2001 |
| Wang X | 20 | 35 | 0.952381 | 1282 | 43 | 2002 |
| Zhang Y | 14 | 23 | 0.7 | 591 | 42 | 2003 |
| Wang J | 19 | 33 | 1 | 1124 | 42 | 2004 |
| Arendt-Nielsen L | 17 | 30 | 0.7727273 | 936 | 40 | 2001 |
| Anand P | 23 | 33 | 1.0454545 | 3166 | 36 | 2001 |
| Kim J | 16 | 26 | 0.7272727 | 741 | 36 | 2001 |
| Chen J | 17 | 27 | 0.7727273 | 783 | 34 | 2001 |
| Kim H | 17 | 28 | 0.7727273 | 819 | 34 | 2001 |
| Li Y | 11 | 18 | 0.5238095 | 381 | 32 | 2002 |
| Kim Y | 16 | 29 | 0.7272727 | 866 | 31 | 2001 |
| Kim S | 15 | 28 | 0.6818182 | 785 | 30 | 2001 |
| Liu Y | 12 | 17 | 0.6315789 | 346 | 29 | 2004 |
| Zhang X | 10 | 20 | 0.4545455 | 408 | 27 | 2001 |
| Wang H | 14 | 25 | 0.6363636 | 1075 | 26 | 2001 |
| Wang S | 13 | 22 | 0.65 | 519 | 25 | 2003 |
| Li H | 12 | 18 | 0.6315789 | 328 | 24 | 2004 |
| Liu X | 9 | 15 | 0.4285714 | 265 | 24 | 2002 |

Citation indices (H-index, g-index, m-index); total citation (TC); publication start year (PSY).
