# Supplementary material for: Capsaicin: A Two-Decade Systematic Review of Global Research Output and Recent Advances Against Human Cancer
Source: Front Oncol. 2022 Jul 13;12:908487. doi: 10.3389/fonc.2022.908487 (PMC9326111; doi:10.3389/fonc.2022.908487)
Supplement: Supplementary Table 5 — Top 20 most cited research articles on capsaicin from 2001 to 2021 based on total citations (TC) and total citation per year (TC/Year). [file Table_5.docx]

| S/N | Title | Journal | TC | TC/Year | Cluster | Reference |
| --- | --- | --- | --- | --- | --- | --- |
| 1 | Bradykinin and nerve growth factor release the capsaicin receptor from PtdIns(4,5)P2-mediated inhibition. | Nature | 1048 | 47.64 | Blue, Green | (1) |
| 2 | An endogenous capsaicin-like substance with high potency at recombinant and native vanilloid VR1 receptors. | Proceedings of the National Academy of Sciences of the United States of America | 713 | 33.95 | Green | (2) |
| 3 | cAMP-dependent protein kinase regulates desensitization of the capsaicin receptor (VR1) by direct phosphorylation. | Neuron | 437 | 20.81 | Green | (3) |
| 4 | A modular PIP2 binding site as a determinant of capsaicin receptor sensitivity | Science | 412 | 20.60 | Blue, Green | (4) |
| 5 | Protein kinase C activation potentiates gating of the vanilloid receptor VR1 by capsaicin, protons, heat and anandamide | The Journal of Physiology | 400 | 18.18 | Green, Red | (5) |
| 6 | Direct phosphorylation of capsaicin receptor VR1 by protein kinase C epsilon and identification of two target serine residues | Journal of Biological Chemistry | 397 | 18.90 | Red | (6) |
| 7 | Potentiation of capsaicin receptor activity by metabotropic ATP receptors as a possible mechanism for ATP-evoked pain and hyperalgesia | Proceedings of the National Academy of Sciences of the United States of America | 384 | 17.45 | Red | (7) |
| 8 | Topical capsaicin for pain management: therapeutic potential and mechanisms of action of the new high-concentration capsaicin 8% patch | British Journal of Anaesthesia | 381 | 31.75 | Blue | (8) |
| 9 | Genistein, EGCG, and capsaicin inhibit adipocyte differentiation process via activating AMP-activated protein kinase | Biochemical and Biophysical Research Communications | 375 | 20.83 | Red | (9) |
| 10 | Increased capsaicin receptor TRPV1-expressing sensory fibres in irritable bowel syndrome and their correlation with abdominal pain | Gut | 323 | 21.53 | Blue, Green | (10) |
| 11 | Protein kinase C phosphorylation sensitizes but does not activate the capsaicin receptor transient receptor potential vanilloid 1 (TRPV1) | Proceedings of the National Academy of Sciences of the United States of America | 315 | 15.75 | Red | (11) |
| 12 | Protease-Activated Receptor 2 Sensitizes the Capsaicin Receptor Transient Receptor Potential Vanilloid Receptor 1 to Induce Hyperalgesia | The Journal of Neuroscience | 305 | 16.05 | Red | (12) |
| 13 | Selective Blockade of the Capsaicin Receptor TRPV1 Attenuates Bone Cancer Pain | The Journal of Neuroscience | 269 | 14.94 | Blue, Green | (13) |
| 14 | N-oleoyldopamine, a novel endogenous capsaicin-like lipid that produces hyperalgesia | The Journal of Biological Chemistry | 269 | 13.45 | Blue | (14) |
| 15 | Systematic review of topical capsaicin for the treatment of chronic pain | British Medical Journal | 258 | 13.58 | Blue | (15) |
| 16 | Sensory fibres expressing capsaicin receptor TRPV1 in patients with rectal hypersensitivity and faecal urgency | The Lancet | 249 | 12.45 | Green | (16) |
| 17 | Forty years in capsaicin research for sensory pharmacology and physiology | Neuropeptides | 244 | 12.84 | Green | (17) |
| 18 | Capsaicin (TRPV1 Agonist) therapy for pain relief: farewell or revival? | The Clinical Journal of Pain | 242 | 16.13 | Blue | (18) |
| 19 | Capsaicin, a component of red peppers, inhibits the growth of androgen-independent, p53 mutant prostate cancer cells | Cancer Research | 241 | 14.18 | Red | (19) |
| 20 | Spider toxins activate the capsaicin receptor to produce inflammatory pain | Nature | 240 | 14.12 | Blue, Green | (20) |

References

1. Chuang HH, Prescott ED, Kong H, Shields S, Jordt SE, Basbaum AI, et al. Bradykinin and nerve growth factor release the capsaicin receptor from PtdIns(4,5)P2-mediated inhibition. *Nat 2001 4116840* (2001) 411:957–62. doi: 10.1038/35082088
2. Huang SM, Bisogno T, Trevisani M, Al-Hayani A, De Petrocellis L, Fezza F, Tognetto M, Petros TJ, Krey JF, Chu CJ, et al. An endogenous capsaicin-like substance with high potency at recombinant and native vanilloid VR1 receptors. *Proc Natl Acad Sci* (2002) 99:8400–8405. doi: 10.1073/pnas.122196999
3. Bhave G, Zhu W, Wang H, Brasier D., Oxford GS, Gereau RW. cAMP-dependent protein kinase regulates desensitization of the capsaicin receptor (VR1) by direct phosphorylation. *Neuron* (2002) 35:721–731. doi: 10.1016/S0896-6273(02)00802-4
4. Prescott ED, Julius D. A modular PIP 2 binding site as a determinant of capsaicin receptor sensitivity. *Science* (2003) 300:1284–1288. doi: 10.1126/science.1083646
5. Vellani V, Mapplebeck S, Moriondo A, Davis JB, McNaughton PA. Protein kinase C activation potentiates gating of the vanilloid receptor VR1 by capsaicin, protons, heat and anandamide. *J Physiol* (2001) 534:813–825. doi: 10.1111/j.1469-7793.2001.00813.x
6. Numazaki M, Tominaga T, Toyooka H, Tominaga M. Direct phosphorylation of capsaicin receptor VR1 by protein kinase Cε and identification of two target serine residues. *J Biol Chem* (2002) 277:13375–13378. doi: 10.1074/jbc.C200104200
7. Tominaga M, Wada M, Masu M. Potentiation of capsaicin receptor activity by metabotropic ATP receptors as a possible mechanism for ATP-evoked pain and hyperalgesia. *Proc Natl Acad Sci U S A* (2001) 98:6951–6956. doi: 10.1073/pnas.111025298
8. Anand P, Bley K. Topical capsaicin for pain management: therapeutic potential and mechanisms of action of the new high-concentration capsaicin 8% patch. *Br J Anaesth* (2011) 107:490–502. doi: 10.1093/bja/aer260
9. Hwang J-T, Park I-J, Shin J-I, Lee YK, Lee SK, Baik HW, Ha J, Park OJ. Genistein, EGCG, and capsaicin inhibit adipocyte differentiation process via activating AMP-activated protein kinase. *Biochem Biophys Res Commun* (2005) 338:694–699. doi: 10.1016/j.bbrc.2005.09.195
10. Akbar A, Yiangou Y, Facer P, Walters JRF, Anand P, Ghosh S. Increased capsaicin receptor TRPV1-expressing sensory fibres in irritable bowel syndrome and their correlation with abdominal pain. *Gut* (2008) 57:923–929. doi: 10.1136/gut.2007.138982
11. Bhave G, Hu H-J, Glauner KS, Zhu W, Wang H, Brasier DJ, Oxford GS, Gereau RW. Protein kinase C phosphorylation sensitizes but does not activate the capsaicin receptor transient receptor potential vanilloid 1 (TRPV1). *Proc Natl Acad Sci* (2003) 100:12480–12485. doi: 10.1073/pnas.2032100100
12. Amadesi S. Protease-activated receptor 2 sensitizes the capsaicin receptor transient receptor potential vanilloid receptor 1 to induce hyperalgesia. *J Neurosci* (2004) 24:4300–4312. doi: 10.1523/JNEUROSCI.5679-03.2004
13. Ghilardi JR. Selective blockade of the capsaicin receptor trpv1 attenuates bone cancer pain. *J Neurosci* (2005) 25:3126–3131. doi: 10.1523/JNEUROSCI.3815-04.2005
14. Chu CJ, Huang SM, De Petrocellis L, Bisogno T, Ewing SA, Miller JD, Zipkin RE, Daddario N, Appendino G, Di Marzo V, et al. N-Oleoyldopamine, a novel endogenous capsaicin-like lipid that produces hyperalgesia. *J Biol Chem* (2003) 278:13633–13639. doi: 10.1074/jbc.M211231200
15. Mason L, Moore RA, Derry S, Edwards JE, McQuay HJ. Systematic review of topical capsaicin for the treatment of chronic pain. *BMJ* (2004) 328:991. doi: 10.1136/bmj.38042.506748.EE
16. Chan C, Facer P, Davis J, Smith G, Egerton J, Bountra C, Williams N, Anand P. Sensory fibres expressing capsaicin receptor TRPV1 in patients with rectal hypersensitivity and faecal urgency. *Lancet* (2003) 361:385–391. doi: 10.1016/S0140-6736(03)12392-6
17. Szolcsányi J. Forty years in capsaicin research for sensory pharmacology and physiology. *Neuropeptides* (2004) 38:377–384. doi: 10.1016/j.npep.2004.07.005
18. Knotkova H, Pappagallo M, Szallasi A. Capsaicin (TRPV1 agonist) therapy for pain relief. *Clin J Pain* (2008) 24:142–154. doi: 10.1097/AJP.0b013e318158ed9e
19. Mori A, Lehmann S, O’Kelly J, Kumagai T, Desmond JC, Pervan M, McBride WH, Kizaki M, Koeffler HP. Capsaicin, a component of red peppers, inhibits the growth of androgen-independent, p53 mutant prostate cancer cells. *Cancer Res* (2006) 66:3222–3229. doi: 10.1158/0008-5472.CAN-05-0087
20. Siemens J, Zhou S, Piskorowski R, Nikai T, Lumpkin EA, Basbaum AI, King D, Julius D. Spider toxins activate the capsaicin receptor to produce inflammatory pain. *Nature* (2006) 444:208–212. doi: 10.1038/nature05285
