# Supplementary material for: Capsaicin: A Two-Decade Systematic Review of Global Research Output and Recent Advances Against Human Cancer
Source: Front Oncol. 2022 Jul 13;12:908487. doi: 10.3389/fonc.2022.908487 (PMC9326111; doi:10.3389/fonc.2022.908487)
Supplement: Supplementary Table 6 — Twenty top leading and most cited countries of capsaicin publications from 2001 to 202. [file Table_6.docx]

| Rank | Countries | Articles | % of 3753 | Freq. | SCP | MCP | MCP/P | TC | AAC |
| --- | --- | --- | --- | --- | --- | --- | --- | --- | --- |
| 1 | USA | 689 | 18.36 | 0.19 | 590 | 99 | 0.1437 | 28869 | 41.9 |
| 2 | China | 546 | 14.55 | 0.15 | 484 | 62 | 0.1136 | 8621 | 15.79 |
| 3 | Japan | 354 | 9.43 | 0.10 | 326 | 28 | 0.0791 | 8578 | 24.23 |
| 4 | Korea | 209 | 5.57 | 0.06 | 178 | 31 | 0.1483 | 5813 | 27.81 |
| 5 | India | 172 | 4.58 | 0.05 | 168 | 4 | 0.0233 | 3171 | 18.44 |
| 6 | Germany | 169 | 4.50 | 0.05 | 130 | 39 | 0.2308 | 3757 | 22.23 |
| 7 | UK | 142 | 3.78 | 0.04 | 113 | 29 | 0.2042 | 7857 | 55.33 |
| 8 | Italy | 109 | 2.90 | 0.03 | 82 | 27 | 0.2477 | 3558 | 32.64 |
| 9 | Brazil | 96 | 2.56 | 0.03 | 75 | 21 | 0.2188 | 1485 | 15.47 |
| 10 | Mexico | 92 | 2.45 | 0.03 | 78 | 14 | 0.1522 | 1390 | 15.11 |
| 11 | Turkey | 90 | 2.40 | 0.03 | 85 | 5 | 0.0556 | 843 | 9.37 |
| 12 | Hungary | 82 | 2.18 | 0.02 | 74 | 8 | 0.0976 | 1730 | 21.1 |
| 13 | Denmark | 79 | 2.10 | 0.02 | 51 | 28 | 0.3544 | 2002 | 25.34 |
| 14 | Australia | 69 | 1.84 | 0.02 | 56 | 13 | 0.1884 | 1742 | 25.25 |
| 15 | Spain | 69 | 1.84 | 0.02 | 64 | 5 | 0.0725 | 1740 | 25.22 |
| 16 | Canada | 52 | 1.39 | 0.01 | 39 | 13 | 0.25 | 1005 | 19.33 |
| 17 | Thailand | 46 | 1.23 | 0.01 | 41 | 5 | 0.1087 | 683 | 14.85 |
| 18 | Sweden | 42 | 1.12 | 0.01 | 35 | 7 | 0.1667 | 1106 | 26.33 |
| 19 | Netherlands | 38 | 1.01 | 0.01 | 23 | 15 | 0.3947 | 1438 | 37.84 |
| 20 | Iran | 37 | 0.99 | 0.01 | 33 | 4 | 0.1081 | 365 | 9.86 |

Countries are classified on the basis of single country publications (SCP), multiple country publications (MCP), and multiple country publications per publication (MCP/P). TC-Total citations; AAC-Average article citations. Korea (North Korea and South Korea).
