# Supplementary figures and images for: Capsaicin: A Two-Decade Systematic Review of Global Research Output and Recent Advances Against Human Cancer
Source: Front Oncol. 2022 Jul 13;12:908487. doi: 10.3389/fonc.2022.908487 (PMC9326111; doi:10.3389/fonc.2022.908487)

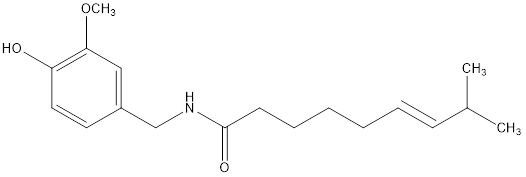

Supplement: Supplementary Figure 1 — Chemical structure of capsaicin. [file Image_1.tif]

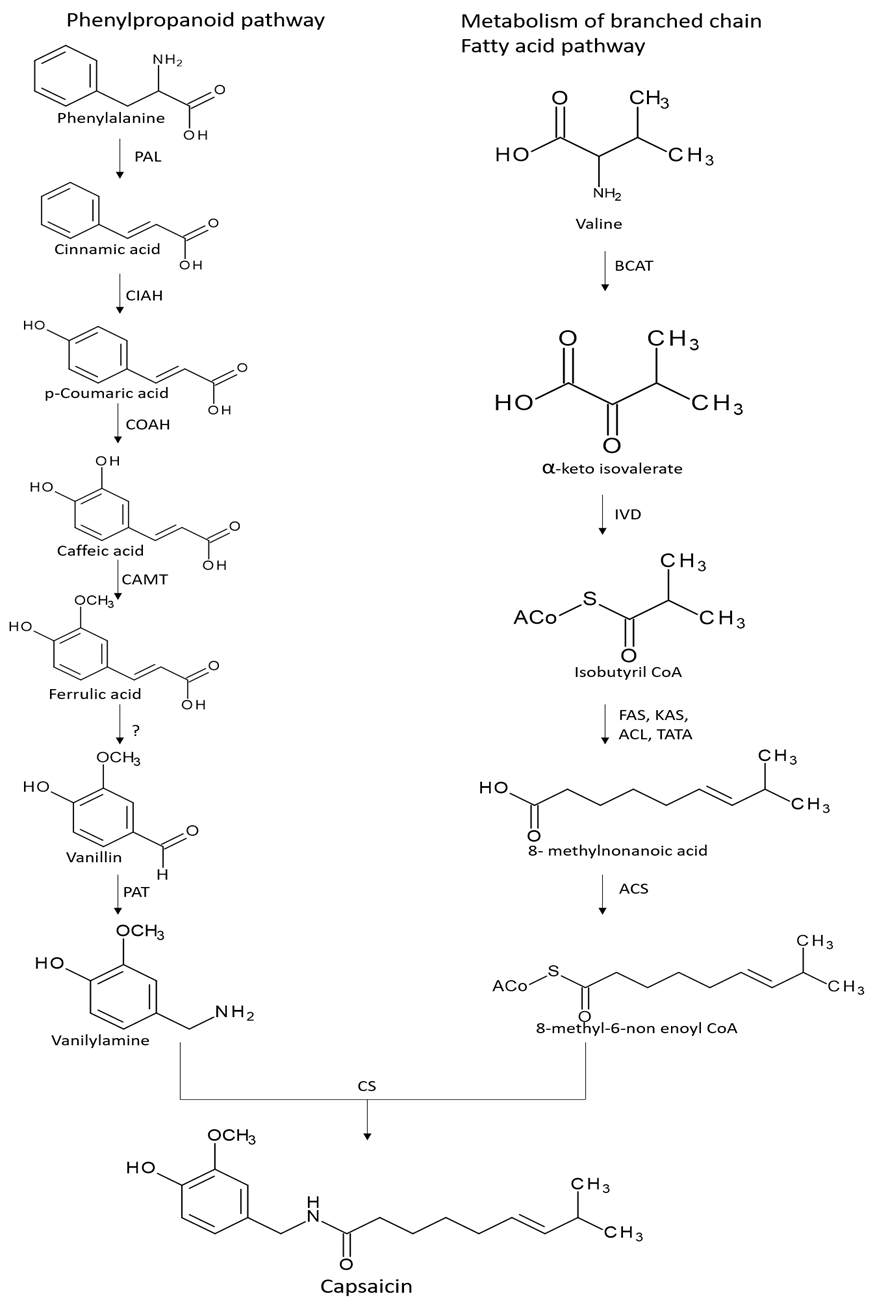

Supplement: Supplementary Figure 2 — Capsaicin biosynthetic pathways (30, 174). PAL-phenylalanine ammonia lyase, CIAH-cinnamic acid 4-hydroxylase, COAH-coumaric acid 3- hydroxylase, CAMT-caffeic acid O-methyltransferase, PAT-putative aminotransferase, BCAT- branched chain amino acid transferase, IVD- isovalerate deshidrogenase, FAS-fatty acid synthase complex, KAS-β-ketoacyl-[acyl-carrier-protein] (ACP) synthase, ACL-acyl carrier protein, TATA- acyl-ACP thioesterase, ACS-acyl-CoA synthase, CS-capsaicin synthase. [file Image_2.tif]

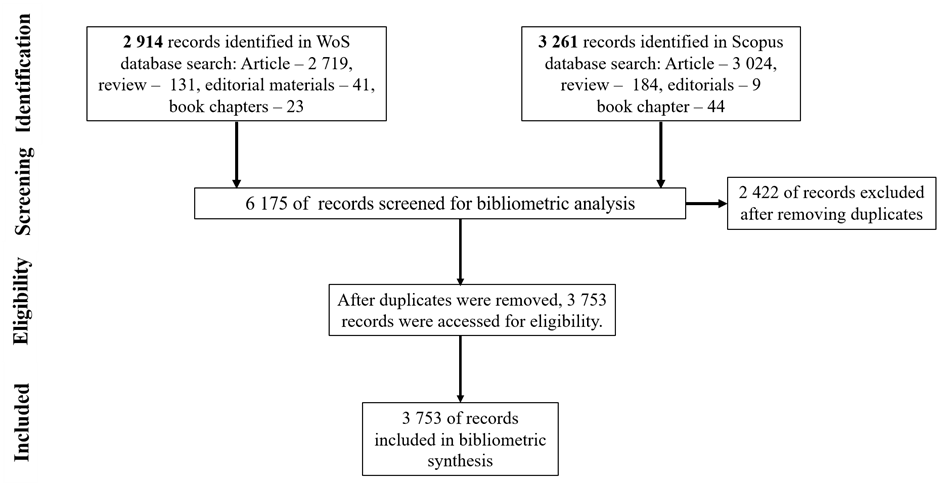

Supplement: Supplementary Figure 3 — Flow chart of the bibliometric study of capsaicin showing the selection and inclusion criteria from Web of Science (WoS) and Scopus databases. [file Image_3.tif]

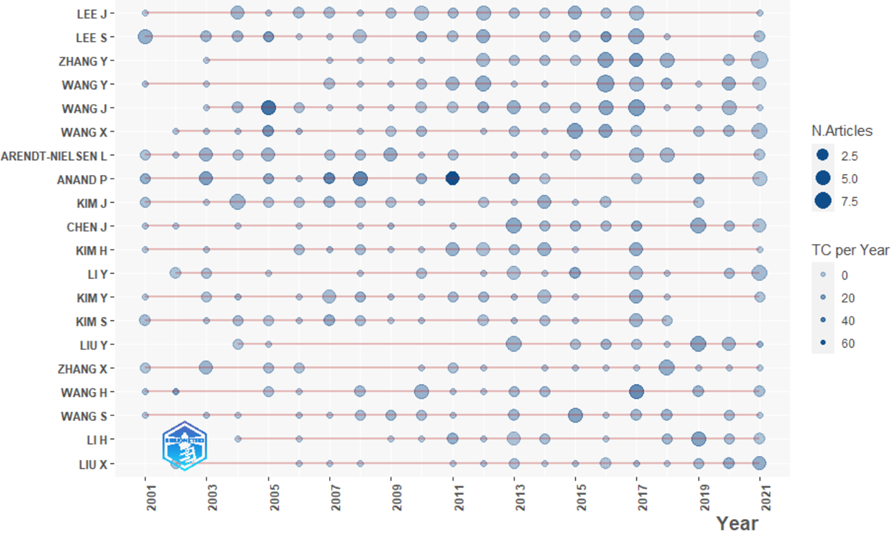

Supplement: Supplementary Figure 4 — Twenty most productive authors in capsaicin research (2001–2021). [file Image_4.tif]

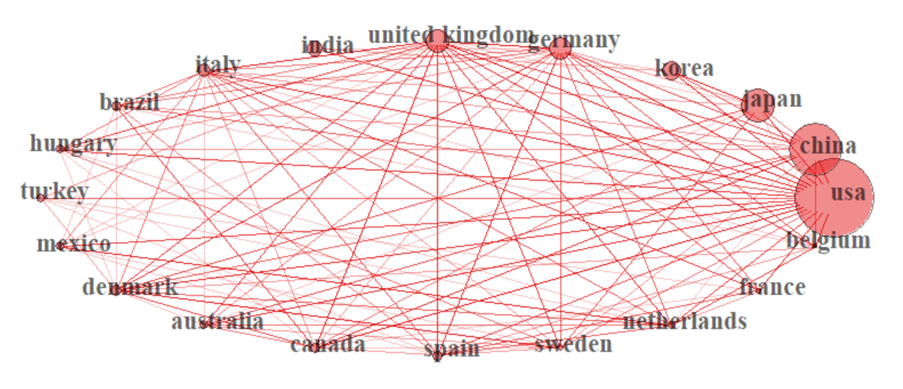

Supplement: Supplementary Figure 5 — Network visualization map showing country collaborations on capsaicin research. The node represents countries. Node diameter signifies the collaboration strength of a country with other countries. Lines depict collaboration pathways between countries. Korea (North Korea and South Korea). [file Image_5.tif]
